# Determination of Seven Human Milk Oligosaccharides (HMOs) in Infant Formula and Adult Nutritionals: First Action 2022.07

**DOI:** 10.1093/jaoacint/qsae001

**Published:** 2024-01-13

**Authors:** Thierry Bénet, Nathalie Frei, Véronique Spichtig, Denis Cuany, Sean Austin

**Affiliations:** Nestlé Institute of Food Safety and Analytical Sciences, Société des Produits Nestlé S.A., Vers-Chez-Les-Blanc, 1000 Lausanne, Switzerland; Nestlé Institute of Food Safety and Analytical Sciences, Société des Produits Nestlé S.A., Vers-Chez-Les-Blanc, 1000 Lausanne, Switzerland; Nestlé Institute of Food Safety and Analytical Sciences, Société des Produits Nestlé S.A., Vers-Chez-Les-Blanc, 1000 Lausanne, Switzerland; Nestlé Institute of Food Safety and Analytical Sciences, Société des Produits Nestlé S.A., Vers-Chez-Les-Blanc, 1000 Lausanne, Switzerland; Nestlé Institute of Food Safety and Analytical Sciences, Société des Produits Nestlé S.A., Vers-Chez-Les-Blanc, 1000 Lausanne, Switzerland

## Abstract

**Background:**

Human milk oligosaccharides (HMOs) are important components of breast milk and may be responsible for some of the benefits of breastfeeding, including resistance to infections and the development of a healthy gut microbiota. Selected HMOs are now available for addition to infant formula, and suitable methods to control the dosing rate are needed.

**Objective:**

To develop and validate a suitable method for the analysis of HMOs in infant formula.

**Method:**

A method was developed for the determination of seven human milk oligosaccharides (2′-fucosyllactose, 3-fucosyllactose, 3′-sialyllactose, 6′-sialyllactose (6′SL), 2′,3-difucosyllactose, lacto-*N*-tetraose (LNT), lacto-*N*-neotetraose (LNnT)) in infant formula and adult nutritionals. The oligosaccharides are labeled at their reducing end with 2-aminobenzamide, separated by liquid chromatography and detected using a fluorescence detector. Maltodextrins are enzymatically hydrolyzed before analysis to prevent potential interference; likewise, an optional β-galactosidase treatment can be used to remove β-galactooligosaccharides. Fructooligosaccharides or polydextrose do not generally interfere with the analysis.

**Results:**

The method has been validated in a single laboratory on infant formula and adult nutritionals. The seven HMOs were spiked into eight matrixes at three or four spike levels, giving a total of 176 data points. Recoveries were in the range of 90.9–109% in all cases except at the lowest spike level in one matrix (elemental formula), where the LNT recovery was 113%, the LNnT recovery was 111%, and the 6′SL recovery was 121%. Relative repeatabilities (RSD(r)) were in the range of 0.1–4.2%. The performance is generally within the requirements outlined in the *Standard Method Performance Requirements* (SMPR^®^) published by AOAC INTERNATIONAL.

**Conclusions:**

The method developed is suitable for the determination of seven HMOs in infant formula and demonstrated good performance during single-laboratory validation.

**Highlights:**

A method has been developed that is suitable for the determination of seven HMOs in infant formula.

Human milk oligosaccharides (HMOs) are the fourth most abundant component of human milk after water, lactose, and fat. They are nondigestible and are believed to be important for the development of a healthy gut microbiota, protecting the infant from infections and for the cognitive development of the infant (1–6). More than 160 different HMOs have been identified to date (7), but quantitative data are available for only around 30 (8). Until recently, HMOs have not been available for addition to infant formula since affordable commercial production of the ingredients was not possible. However, developments in biotechnology have overcome this problem, and formula manufacturers have started to introduce HMOs in their formula (9). The first commercial formula to be launched containing an HMO contained 2′-fucosyllactose (2′FL). This was closely followed by the launch of formula containing 2′FL and lacto-*N*-neotetraose (LNnT). Ingredient suppliers have continued to develop further HMOs, and it is expected that the seven HMOs covered by this method will soon be found in formula from different manufacturers.

Many methods for the determination of the oligosaccharides in human milk have been published, including methods based on high-performance anion exchange chromatography with pulsed amperometric detection (HPAEC-PAD) (10, 11), liquid chromatography–mass spectrometry (LC-MS) (12, 13), LC with fluorescence detection (LC-FLD) (14–16), and capillary electrophoresis with laser-induced fluorescence detection (CE-LIF) (17–19). However, direct application of those methods to infant formula may not always be possible, since the infant formula matrix is different and may contain components that are not present in human milk (for example, maltodextrins or fructooligosaccharides). We previously published two methods for the determination of 2′FL and LNnT in infant formula (20) using either HPAEC-PAD or LC-FLD after derivatization of the HMOs with 2-aminobenzamide (2AB). Both methods performed comparably with recoveries in the range of 94 –111% and intermediate reproducibility in the range of 2–8%. Problems were encountered in one matrix, where the HPAEC-PAD method had a poor recovery, whereas the LC-FLD method returned results in line with expectations. This was postulated to be due to non-covalent interactions between analyte and matrix, which were not disrupted when using simple water extraction but may have been disrupted by the conditions used during 2AB labeling (e.g., the use of dimethyl sulfoxide as a solvent). Prieto et al. (21) also published a method for the determination of 2′FL in infant formula by HPAEC-PAD and achieved excellent recoveries (94–102%) and precision (RSD <2%). Ma et al. (22) applied LC-MS for the analysis of five sialylated oligosaccharides naturally present in milk-based formula, including the HMOs 3′-sialyllactose (3′SL) and 6′-sialyllactose (6′SL). They achieved a repeatability of less than 7% for 3′SL and below 5% for 6′SL but did not report any data regarding the method trueness. Christensen et al. (23) applied LC with refractive index (RI) detection for the analysis of 2′FL and 3FL in infant formula. The method achieved recoveries in the range of 88–96% and RSDs below 4%. Seydametova et al. (24) developed an enzyme-coupled reaction using a cloned α-1,2-fucosidase and L-fucose dehydrogenase to develop a colorimetric method for the determination of 2′FL. The authors demonstrated that the enzymes had specificity for α-1,2-linked fucose, and thus could be applied in the absence of any other oligosaccharides containing α-1,2-linked fucose residues, but they did not report method performance data for the analysis of 2′FL in formula.

In this work, we have adapted our method for 2′FL and LNnT in formula (20), expanding the number of analytes and their concentration ranges to meet the requirements set out in the relevant *Standard Method Performance Requirements* (SMPRs^®^): SMPR 2020.003 for 2′FL, SMPR 2020.004 for LNnT, SMPR 2021.003 for 3′SL, SMPR 2021.004 for 3-fucosyllactose (3FL), SMPR 2021.005 for 6′SL, SMPR 2021.006 for 2′,3-difucosyllactose (DFL), and SMPR 2021.007 for lacto-*N*-tetraose (LNT). The method is based on labeling of the HMO with 2AB, followed by separation by liquid chromatography and fluorescence detection. This technique was introduced by Bigge et al. (25) when they demonstrated quantitative incorporation of 2AB and 2-anthranilic acid (2AA) for oligosaccharide labeling and labeling efficiencies close to 90%. We have since demonstrated the applicability of 2AB labeling for the quantitative determination of galactooligosaccharides (26), oligosaccharides in milk (15), and 2′FL and LNnT in formula (20).

## Single-Laboratory Validation Study

### Materials

- *Blank matrixes.—*An infant formula powder with FOS and GOS and an infant formula elemental powder were acquired from the SPIFAN 2 matrixes kit. An adult nutritional ready-to-feed (RTF) with FOS and GOS, a soy-based infant formula powder, and an infant formula RTF with FOS and GOS were purchased from a local pharmacy. An infant formula powder with partially hydrolyzed protein and probiotics, an infant formula powder (goat milk based), and another infant formula powder with FOS and GOS were acquired from Nestlé Nutrition, Vevey, Switzerland.
- *Matrixes containing HMOs*.—A commercial infant formula powder with GOS and 2′FL and a commercial infant formula RTF with 2′FL were purchased at a local supermarket or pharmacy. Commercial infant formula powder with probiotic and 2′FL, commercial infant formula powder with 2′FL and LNnT, and commercial infant formula powder with 2′FL, LNnT, and GOS were acquired from Nestlé Nutrition, Vevey, Switzerland. A pilot infant formula powder with 2′FL and LNnT was prepared at Nestlé Product Technology Centre, Konolfingen, Switzerland. A pilot infant formula powder with GOS and HMOs (laboratory reference sample) was prepared at Nestlé Development Centre Nutrition, Askeaton, Ireland.

### Study Design

Table 1 summarizes the seven SMPRs for the seven HMOs. The validation experiments were designed to test the method against those SMPRs.

**Table 1.** Summary of method performance requirements for HMO analysis

SMPR	HMO	Concn range, mg/100 g^a^	LoQ, mg/100 g^a^	Recovery, %	RSD(r), %	RSD(R), %
2020.003	2′FL	5.0–20	≤4.0	85–110	≤5	≤10
2020.003	2′FL	>20–500	≤4.0	90–110	≤5	≤10
2020.004	LNnT	5.0–20	≤4.0	85–110	≤5	≤10
2020.004	LNnT	>20–100	≤4.0	90–110	≤5	≤10
2021.004	3FL	4.0–20	≤3.2	85–110	≤5	≤10
2021.004	3FL	>20–600	≤3.2	90–110	≤5	≤10
2021.006	DFL	1.5–20	≤1.2	85–110	≤5	≤10
2021.006	DFL	>20–100	≤1.2	90–110	≤5	≤10
2021.007	LNT	2.0–20	≤1.6	85–110	≤5	≤10
2021.007	LNT	>20–300	≤1.6	90–110	≤5	≤10
2021.003	3′SL	1.5–20	≤1.2	85–110	≤5	≤10
2021.003	3′SL	>20–150	≤1.2	90–110	≤5	≤10
2021.005	6′SL	2.0–20	≤1.6	85–110	≤5	≤10
2021.005	6′SL	>20–150	≤1.6	90–110	≤5	≤10

a Concentrations apply to i) “ready-to-feed” liquids “as is,” ii) reconstituted powders (25 + 200 g water), iii) liquid concentrates diluted 1:1 by weight.

Calibration fit was assessed by injecting calibration solutions of each HMO at six concentrations, adapted according to the working range required for each HMO (Table 2); each level was prepared in triplicate. The ratio of the peak area of the HMO/the peak area of the internal standard was plotted against the concentration of the HMO. A linear model forced through the origin was used to fit the data for calibration purposes. The relative residuals were calculated and plotted against the analyte concentration.

**Table 2.** Concentration of HMO solutions used to assess calibration fit

HMO	Level 1, µg/mL	Level 2, µg/mL	Level 3, µg/mL	Level 4, µg/mL	Level 5, µg/mL	Level 6, µg/mL
2′FL	10.3	113	227	350	494	618
3FL	10.5	116	232	358	505	632
DFL	4.33	30.3	60.6	95.3	130	173
LNT	6.90	60.4	121	190	259	345
LNnT	8.98	44.9	89.8	135	180	224
3′SL	3.24	30.4	64.8	105	146	194
6′SL	3.73	35.0	74.7	121	168	224

Limits of detection (LoD) and quantification (LoQ) were estimated by spiking the HMOs at approximately 10% of the desired LoQ into blank formula (four different matrixes) and water (*see*Supplemental Table 1). The spiked samples were analyzed 10 times each to establish a mean and standard deviation for each. The LoD and LoQ were then estimated following Equations 1 and 2 (27). The milk-based formula contained native levels of 3′SL and 6′SL, which were too high to allow the LoD and LoQ estimation in those matrixes. Therefore, the estimation of LoD and LoQ for 3′SL and 6′SL could only be estimated from the spikes in water and soy-based formula.

(1) $$\mathrm{LoD}\;=\;\bar{C_{\mathrm{HMO}}}+{SD}_{\mathrm{HMO}}\times 3$$

(2) $$\mathrm{LoQ}\;=\;\bar{C_{\mathrm{HMO}}}+{SD}_{\mathrm{HMO}}\times 10$$

where $\bar{C_{\mathrm{HMO}}}$= the mean concentration of HMO determined in the 10 replicates; SD_HMO_ = the standard deviation of the mean HMO concentration determined in the 10 replicates.

Recovery was assessed by spiking eight blank matrixes with the seven HMOs at four different concentrations covering the desired range for each HMO specified in the SMPRs (*see*Supplemental Table 2). Spiking was performed by adding concentrated solutions of the HMOs or adding powdered HMOs for high concentration levels. If the matrixes were powders, they were reconstituted before spiking. If RTF, the matrixes were spiked directly. After spiking, the samples were homogenized, aliquoted, and stored frozen until analysis. None of the matrixes contained native levels of 2′FL, 3FL, DFL, LNT, or LNnT; therefore, the recovery could be calculated according to Equation 3. Most of the matrixes contained native levels of 3′SL and 6′SL; therefore, the recovery was calculated after subtracting the amount of the HMO measured in the non-spiked sample according to Equation 4.

(3) $$\mathrm{Rec}=\frac{C_{m}}{C_{S}}\times 100$$

(4) $$\mathrm{Rec}=\frac{{(C}_{m}-C_{n})}{C_{S}}\times 100$$

where Rec = the spike recovery expressed in %; C_m_ = the concentration of HMO measured in the spiked sample in mg/100 g; C_S_ = the concentration of HMO spiked into the sample in mg/100 g; C_n_ = the native concentration of HMO in the non-spiked sample in mg/100 g.

Repeatability (r) and intermediate reproducibility (iR) were assessed by analyzing the eight spiked samples and seven commercial or pilot plant samples in duplicate on at least six different days by four different operators on three different instruments. The in-house statistical package Q-Stat was used to calculate the robust SD_rob_(r) and SD_rob_(iR) using Equations 5 and 6.

(5) $${SD}_{\mathrm{rob}}\left(r\right)=1.0484\times \begin{array}{c} Med\\ i=1,..n \end{array}\left\{\left|x_{i1}-x_{i2}\right|\right\}$$

(6) $${SD}_{\mathrm{rob}}(iR)=\sqrt{{SD}_{\mathrm{rob}}^{2}\left(b\right)+\frac{1}{2}\times {SD}_{\mathrm{rob}}^{2}(r)}$$

where *n* is the number of (single or duplicate) determinations; *x_i_* is the individual result within the set of single determinations with *i* going from 1 to *n*; *x*_*i*1_ and *x*_*i*2_ are the two results within the set of duplicate determination with i going from 1 to n; *SD_rob_*(b) is the robust standard deviation between the means of duplicates; *SD_rob_*(*r*) is the robust standard deviation of repeatability; and *SD_rob_*(*iR*) is the robust standard deviation of intermediate reproducibility.

**AOAC *Official Method*^SM^ 2022.07**

**Seven Human Milk Oligosaccharides in Infant Formula and Adult Nutritionals**

**HILIC-FLD After 2AB Labeling**

**First Action 2022**

[Applicable to the determination of 2′-fucosyllactose (5–500 mg/100 g), 3-fucosyllactose (4–600 mg/100 g), 2′,3-difucosyllactose (1.5–100 mg/100 g), 3′-sialyllactose (1.5–150 mg/100 g), 6′-sialyllactose (2–150 mg/100 g), lacto-*N*-tetraose (2–300 mg/100 g), and lacto-*N*-neotetraose (5–100 mg/100 g) in infant formula and adult nutritionals. *Limitations*: For soy-based formula, the working range for 6′-sialyllactose is different (4–150 mg/100 g). For elemental formula, DFL, LNT, and LNnT may be overestimated at concentrations close to limit of quantification (LoQ). For formula containing polydextrose, the accurate determination of 2′,3-difucosyllactose may not be possible at the lower end of the working range.]

*Caution*: The method employs corrosive, irritant, and flammable chemicals. Before use, refer to safety data sheets (SDS) for all chemicals, identify risks, and take appropriate safety precautions including (but not limited to) use of lab coat, safety glasses, and gloves. Work in a fumehood when handling acids, ammonia solution, and 2-picoline borane. Ensure waste disposal is in accordance with local requirements. Some individuals are allergic to the amyloglucosidase enzyme used in this method. Allergic individuals should not handle the powdered enzyme; they should find another analyst to prepare the working solution and take appropriate precautions when handling the enzyme in solution.

### A. Principle

Samples are reconstituted in water, and oligosaccharides (OS) present in samples are extracted at 70°C. Aliquots of the diluted sample are taken and treated with amyloglucosidase to hydrolyze any maltooligosaccharides present. A separate aliquot is additionally treated with β-galactosidase if the sample contains GOS. An internal standard (laminaritriose) is added, and the oligosaccharides are fluorescently labeled with 2-aminobenzamide (2AB). Labeled extracts are diluted with acetonitrile before injection on an ultrahigh-performance liquid chromatography system with fluorescence detector (UHPLC-FLD) equipped with a hydrophilic interaction liquid chromatography (HILIC) analytical column. The analytes are separated using a gradient of aqueous ammonium formate in acetonitrile and detected with a fluorescence detector.

### B. Apparatus

- *Microcentrifuge tubes 1.5 mL*.—Safe lock or screw cap.
- *Microcentrifuge tubes 2 mL*.—Safe lock or screw cap.
- *Floating rack for microtubes*.
- *Centrifuge*.—For 1.5 and 2 mL microtubes able to operate at 10 000 × *g*.
- *Water bath or heating block*.—For 2 mL tubes able to operate at 65 ± 0.5°C.
- *Water bath*.—With stirring plate able to operate at 70 ± 0.5°C.
- *Vortex mixer*.
- *Pipet dispenser*.—With tips 0.1 mL to 1.0 mL.
- *Analytical balance*.—With a precision of 0.01 g able to weight 1 kg.
- *Analytical balance*.—With a precision of 0.1 mg.
- *Volumetric flasks.*—10 to 1000 mL.
- *Glass tubes.*—10 mL.
- *Autosampler vials.*—2 mL with screw cap PTFE.
- *Ultrasonic bath*.
- *Glass bottles with screw caps.—*250 mL.
- *pH meter.*—Reading to ± 0.1 pH.
- *Micropipets with tips*.—0.02 to 10 mL.
- *UHPLC column.*—ACQUITY UPLC BEH Glycan, 1.7 µm; 2.1 mm × 150 mm (Waters, Millford, MA, USA).
- *Ultrahigh-performance liquid chromatography instrument equipped with a gradient pump*.—Able to deliver a flow of 0.5 mL/min with a backpressure up to 1000 bar, an online degasser, an autosampler equipped with refrigerated sample compartment, a temperature-controlled column compartment able to maintain a stable temperature up to 75 ± 2.0°C, and a fluorescence detector.

### C. Chemicals and Reagents

- *Deionized water*.—18 MΩ.
- *Dimethyl sulfoxide, puriss p.a.*
- *2-aminobenzamide (2AB)*. The 2AB should be a white to off-white crystalline powder. If the 2AB is not white, it is recommended to recrystallize twice from ethanol (95%) to obtain a white crystalline powder before use.
- *2-Methylpyridine borane complex (2-picoline borane) 95%.*
- *Formic acid*.—GR for analysis.
- *Glacial acetic acid anhydrous*.—GR for analysis.
- *Acetonitrile*.—Gradient grade for liquid chromatography.
- *Laminaritriose >90%*.
- *Ammonium hydroxide solution 25–30%.—*GR for analysis.
- *Ammonium acetate*.
- *Sodium hydroxide solution.—*1 M.
- *β-galactosidase (Aspergillus niger)*.—4000 U/mL (e.g., from Megazyme).
- *Amyloglucosidase (Aspergillus niger)*.—9 U/mg (e.g., from Roche Diagnostics, Indianapolis, IN, USA, 11 202 367 001).
- *2′-fucosyllactose (2′FL)*.—With accurately known purity (e.g., from DSM/Glycom, Hørsholm, Denmark).
- *3-Fucosyllactose (3FL)*.—With accurately known purity (e.g., from DSM/Glycom).
- *Difucosyllactose (DFL)*.—With accurately known purity (e.g., from DSM/Glycom).
- *3′-Sialyllactose (3′SL)*.—With accurately known purity (e.g., from DSM/Glycom).
- *6′-Sialyllactose (6′SL)*.—With accurately known purity (e.g., from DSM/Glycom).
- *Lacto-N-tetraose (LNT)*.—With accurately known purity (e.g., from DSM/Glycom).
- *Lacto-N-neotetraose (LNnT)*.—With accurately known purity (e.g., from DSM/Glycom).

### D. Preparation of Reagents

*Note:* The purity and moisture content of the different HMO standards used for calibration must be accurately known to three significant figures. Purity should be checked using a combination of techniques including (but not limited to) quantitative NMR and liquid chromatography. Moisture should be determined by Karl Fischer titration. The concentrations of the standard solutions are calculated with correction of the known purity and moisture. Ideally, standard manufacturers will supply HMO standards in sealed vials and will provide CoA specifying the purity and the moisture content (e.g., DSM-Glycom). Store HMO standards according to the supplier’s recommendations or in a desiccator at room temperature. It is recommended to determine the moisture content by Karl Fischer titration before preparation of stock solutions since the standards may gain moisture during storage.

- *2′FL standard stock solution (10 mg/mL*) .—Into a 5 mL volumetric flask, weigh 50 ± 5 mg of 2′FL standard [**C**(**n**)], and dilute to the volume with water. Store this solution at −18°C for up to 1 year.
- *3FL standard stock solution (10 mg/mL).—*Into a 5 mL volumetric flask, weigh 50 ± 5 mg of 3FL standard [**C**(**o**)], and dilute to the volume with water. Store this solution at −18°C for up to 1 year.
- *DFL standard stock solution (5 mg/mL).—*Into a 5 mL volumetric flask, weigh 25 ± 2.5 mg of DFL standard [**C**(**p**)], and dilute to the volume with water. Store this solution at −18°C for up to 1 year.
- *3′SL standard stock solution (5 mg/mL).—*Into a 5 mL volumetric flask, weigh 25 ± 2.5 mg of 3′SL standard [**C**(**q**)], and dilute to the volume with water. Store this solution at −18°C for up to 1 year.
- *6′SL standard stock solution (5 mg/mL).—*Into a 5 mL volumetric flask, weigh 25 ± 2.5 mg of 6′SL standard [**C**(**r**)], and dilute to the volume with water. Store this solution at −18°C for up to 1 year.
*Note:* 3′SL and 6′SL standards are generally supplied as sodium salts. Convert the concentration of the standard solution into the SL free acid by dividing the weighed amount by the molecular weight of the sodium salt (655.53) and multiplying by the molecular weight of the free acid (633.55).
- *LNT standard stock solution (10 mg/mL).—*Into a 5 mL volumetric flask, weigh 50 ± 5 mg of LNT standard [**C**(**s**)], and dilute to the volume with water. Store this solution at −18°C for up to 1 year.
- *LNnT standard stock solution (10 mg/mL).—*Into a 5 mL volumetric flask, weigh 50 ± 5 mg of LNnT standard [**C**(**t**)], and dilute to the volume with water. Store this solution at −18°C for up to 1 year.
- *Working standard solutions.—*Prepare mixed standard solutions at six different concentrations, by following the scheme in Table 2022.07A and diluting to the final volume in volumetric flasks. If the samples do not contain all of the HMOs, it is possible to calibrate using only the relevant HMOs; the concentrations may also be adapted to the samples being analyzed if the full range is not required. The working standard solutions can be stored for up to 1 week at 4–8°C or up to 1 year at −18°C.
- *Laminaritriose internal standard stock solution (1.0 mg/mL).—*Transfer about 50 mg of laminaritriose [**C**(**h**)] into a 50 mL volumetric flask and complete to the mark with water. This solution can be stored for up to 1 year at −18°C
- *Laminaritriose internal standard working solution (0.4 mg/mL).—*Transfer 10 mL of the laminaritriose stock solution [**D**(**i**)] into a 25 mL volumetric flask and make up to the mark with water. This solution can be stored for up to 1 year at –18°C.
- *Sodium acetate buffer (0.2 M, pH 4.5 ± 0.05*).—Into a beaker containing 400 mL of demineralized water [**C**(**a**)], pipet 5.8 mL of glacial acetic acid [**C**(**f**)]. Adjust to pH 4.5 with sodium hydroxide solution 1 M [**C**(**k**)]. Transfer the solution to a 500 mL volumetric flask and make up to the mark with water. This solution can be stored for up to 1 month at 4–8°C.
- *Amyloglucosidase (AMG) solution (70 U/mL in 0.2 M sodium acetate buffer pH 4.5).—*Weigh into a glass tube an amount of amyloglucosidase [**C**(**m**)] corresponding to 14 ± 2 U of AMG per test sample, and add sodium acetate buffer (0.2 M pH 4.5 [**D**(**k**)]) to reach a final concentration of 70 U/mL. Dissolve by mixing in a vortex mixer. This solution is prepared on the day of use and kept at 4°C until use.
- *Mixed enzyme solution (β-galactosidase + amyloglucosidase).—*Depending on the number of tests to perform (100 µL/test), prepare an 8:2 ratio mixture of AMG (70 U/mL in Na acetate buffer 0.2 mol/L pH 4.5 [**D**(**l**)] and beta-galactosidase enzyme suspension (4000 U/mL) [**C**(**l**)]. This solution is prepared on the day of use and kept at 4°C until use.
- *Water–acetonitrile solution (25:75).—*Add 125 ± 2 mL of water [**C**(**a**)] to 375 ± 2 mL of acetonitrile [**C**(**g**)] in a glass bottle and mix. This solution can be stored for up to 3 months at room temperature.
- *2AB labeling reagent 2AB [0.35 mol/L]–picoline borane [1.0 mol/L] in DMSO–acetic acid [70:30] solution.—*Pipet the volume of dimethyl sulfoxide (DMSO) [**C**(**b**)] and glacial acetic [**C**(**f**)] acid in a 50 mL glass tube according to the number of tests to perform (Table 2022.07B). Mix the solution using a vortex mixer. Weigh the amount of 2-aminobenzamide (2AB) [**C**(**c**)] and picoline borane [**C**(**d**)] (Table 2022.07B) in another 50 mL glass tube, and then add the corresponding volume of DMSO–acetic acid (Table 2022.07B). Mix using a vortex mixer. The labeling reagent solution can be prepared on the day of use or can be stored at –18°C protected from moisture for up to 3 months.

### E. Mobile Phase Preparation

- *Eluent A*.—Acetonitrile [**C**(**g**)].
- *Eluent B*.—*Ammonium formate (50 mM, pH 4.4).—*Add 2.3 g ± 0.1 g (1.89 mL) of formic acid (100%) [**C**(**e**)] into a beaker containing 800 mL of water. Adjust the pH to 4.40 ± 0.05 with ammonium hydroxide solution (25–30%) [**C**(**i**)]. Transfer quantitatively to a 1 L volumetric flask, and dilute to the volume with water. This solution can be stored for up to 10 days at room temperature.

### F. Sample Preparation

- For powder products, reconstitute powder or liquid concentrates according to the instructions. For example, weigh 25 g of infant formula powder into a bottle and add 200 g of water. Place the mixture in a water bath at 70 ± 2°C for 20–25 min under constant stirring. Cool the solution to room temperature.
- For reconstituted products (as prepared above) or products that are sold as ready-to-feed (RTF), weigh an amount of reconstituted product or RTF product into a 50 mL volumetric flask, such that the concentration of HMO in the final volume is within the range of the calibration curve. Suggested dilutions are shown in Table 2022.07C. For viscous RTF products (such as adult nutritional products), high dilutions are recommended. Samples that do not contain GOS can be analyzed using Preparation A [**F**(**c**)] only. Samples containing GOS should be analyzed using both Preparation A [**F**(**c**)] and Preparation B [**F**(**d**)].
- *Preparation A (for all samples*).—Using a micropipet, transfer 200 µL of diluted sample [**F**(**b**)] into a 1.5 mL microtube. To each tube add AMG solution [**D**(**l**)] (100 µL). Mix using a vortex mixer, and place tubes in a water bath at 60 ± 2°C for 60 ± 2 min. Cool the samples and briefly spin in a centrifuge (4000 × *g*, 5–10 s) to remove drops from the lid. Add laminaritriose working solution [**D**(**j**)] (0.4 mg/mL, 100 µL), mix using a vortex mixer, and continue from step [**F**(**g**)].
- *Preparation B (only for samples containing GOS*).—Using a micropipet, transfer 200 µL of diluted sample [**F**(**b**)] into a 1.5 mL microtube. To each tube add mixed enzyme solution [**D**(**m**)] (100 µL). Mix using a vortex mixer, and then place tubes in a water bath at 60 ± 2°C for 60 ± 2 min. Cool the samples and briefly spin in a centrifuge (4000 × *g*, 5–10 s) to remove drops from the lid. Add laminaritriose working solution [**D**(**j**)] (0.4 mg/mL, 100 µL), mix using a vortex mixer, and continue from step [**F**(**g**)].
- Prepare a reagent blank by adding 200 µL of water to a 1.5 mL microtube. Prepare in the same way as samples from step [**F**(**c**)]. If using preparation B, prepare two reagent blanks with one following Preparation A [**F**(**c**)] and the other following Preparation B [**F**(**d**)] and continue from step [**F**(**g**)].
- Prepare the calibration standards by transferring 200 µL of each of the working standard solutions [**D**(**h**)] into separate 1.5 mL microtubes. To each, add sodium acetate buffer [**D**(**k**)] (0.2 M, pH 4.5, 100 µL) and laminaritriose working solution [**D**(**j**)] (0.4 mg/mL, 100 µL) and continue from step [**F**(**g**)].
- Transfer 100 µL of sample [**D**(**c**)] or [**D**(**d**)], reagent blank [**D**(**e**)], or standard [**D**(**f**)] to a 2.0 mL microtube and add 100 µL of 2AB labeling reagent [**D**(**o**)]. Seal the tubes, mix in a vortex mixer, and place in a water bath at 65 ± 2°C for 60 ± 5 min.
- Place the tubes in a refrigerator or an ice bath for 5 min to cool.
- To each tube add 1.0 mL of water–acetontitrile (25:75) solution [**D**(**n**)] and mix in a vortex mixer.
- Spin the tubes on a centrifuge (10 000 × *g*, 5 min) to remove particles, and transfer around 800–900 µL of the supernatant to a vial suitable for the LC autosampler.

### G. Chromatographic Conditions

The UHPLC system is equipped with an ACQUITY UPLC BEH Glycan column (2.1 mm × 150 mm, 1.7 µm) or (preferably) an ACQUITY UPLC BEH Premier Glycan column (2.1 mm × 150 mm, 1.7 µm). The column is held at 60 ± 2°C, and the injection volume is 2 µL. The analytes are separated using the gradient described in Table 2022.07D, and they are detected by means of a fluorescence detector (excitation λ = 330 nm, emission λ = 420 nm). If samples based on goat or sheep milk or elemental formula are to be analyzed for 6′SL, those samples should be injected a second time at a column temperature of 75 ± 2°C, for the determination of 6′SL. The calibration standards should also be injected a second time at 75°C. In general, if matrix components interfere with the analysis of one of the HMOs, using a column temperature of 75°C will often remedy the issue. However, at 75°C 3FL will co-elute with lactose-phosphate, so 3FL cannot be analyzed under such conditions.

### H. System Suitability Check

Run the LC system under the initial conditions and ensure the system pressure and detector baseline are stable. Inject the standard with the highest concentration. The height of the tallest HMO peak should reach between 50 and 90% of the full scale. Adjust the detector settings if this is not the case. Check that LNT, LNnT, and 6′SL are well resolved from one another (resolution should be ≥1.6). If desired, a sample of cow’s milk can be analyzed with and without a spike of 2′FL; prepare the milk as a “ready to feed” sample. A small peak of lactose phosphate should be visible after the peak of 2′FL. The 2′FL and lactose phosphate should be separated with a resolution ≥1.6. If this is not the case, the column temperature can be adapted by ±5°C. After changing temperature, check the resolution of the 2′FL and lactose phosphate as well as the resolution of LNT, LNnT, and 6′SL (if these analytes are to be included in the analysis). Example chromatograms of the HMO standards spiked into bovine milk and of the seven HMOs in an infant formula are shown in Figure 2022.07A. Inject 3–5 times one of the standards and check that the peak areas are stable. Pay particular attention to 6′SL if it is being analyzed. If the peak area is not stable, it may be necessary to condition the column with fetuin. For fetuin conditioning, prepare a 10 mg/mL solution of fetuin, and make at least three injections (1 µL) of the fetuin solution running the method for HMO analysis. Repeat the injections of HMO standards to see if the peak areas are stable (repeat until stability is achieved).

### I. Calibration and Calculations

It is recommended to use bracketed calibration, injecting six standards followed by a maximum of 30 samples, and then six standards, etc. Use the instrument software to plot a six-point calibration curve of $\frac{\mathrm{Area}\;\mathrm{HMO}}{\mathrm{Area}\;IS}$ against the concentration of HMO in µg/mL, forcing the line through the origin.

For samples that do not contain GOS, the concentration of all HMOs can be determined from the results of preparation A [F(c)].

For samples containing GOS: Concentrations of 2′FL, 3FL, LNT, and LNnT should be determined from the results of preparation A [F(c)]. DFL, 3′SL, and 6′SL should be determined from the results of preparation B [F(d)].

For samples based on sheep milk or goat milk, or for elemental formula, follow the above guidelines, but 6′SL should be determined after running the analysis an additional time with the column temperature at 75°C.

The concentration (C_D_) of the diluted sample is read from the calibration curve.

The concentration of HMO in the ready-to-feed sample (or in reconstituted powders) is calculated according to Equation 7.

(7) $$C_{\mathrm{RTF}}=C_{D}\times \frac{V}{m_{s}}\times 0.1$$

where C_RTF_ = concentration of HMO in ready-to-feed sample or reconstituted sample in mg/100 g; C_D_ = concentration of HMO in the diluted sample read from the calibration curve in µg/mL; V = volume to which the sample was diluted in mL; m_s_ = mass of sample diluted to volume V in g; and 0.1 = factor to convert µg/g to mg/100 g.

For powdered products, the concentration of HMO in the powders can be calculated using Equation 8.

(8) $$C_{p}=\;C_{\mathrm{RTF}}\times \frac{\left(m_{p}+\;m_{w}\right)}{m_{p}}$$

where C_p_ = concentration of HMO in powder samples in mg/100 g; C_RTF_ = concentration of HMO in the reconstituted sample in mg/100 g (as calculated above); m_p_ = mass of powder used to prepare reconstituted sample in g; and m_w_ = mass of water used to prepare reconstituted sample in g.

## Results

### Method Development

We previously developed a method for the determination of oligosaccharides in human milk (15) and a method for the determination of 2′FL and LNnT in infant formula (20). In both methods, the HMOs were derivatized with 2AB, as described previously for *N*-glycan analysis (25). The label is required for detection of the oligosaccharides that do not contain a chromophore or fluorophore and has been demonstrated to be incorporated in a quantitative manner (25). It also introduces a certain amount of selectivity since the oligosaccharides containing an aldose at the reducing end are derivatized, whereas those with a ketose at the reducing end and non-reducing oligosaccharides are not derivatized. The method developed here follows the same principle as the previous methods; however, the conditions for 2AB labeling have been modified, replacing the reducing agent sodium cyanoborohydride with picoline borane (28, 29). Picoline borane has several advantages over sodium borohydride; it is less toxic (29), the reaction can be carried out in aqueous conditions (28), and the reaction time is shorter (one hour instead of two).

After labeling with 2AB, the excess reagents used for the reaction are normally removed before chromatographic analysis. In our previous method, the excess reagents were removed by loading the sample on the guard column and washing away the excess reagents before switching the guard in line with the analytical column for the analysis (15, 20, 30). However, when running with UHPLC and using small injection volumes, it is possible to avoid this step, thus simplifying the method and the equipment requirements. Although everything is injected on the column, it is possible to achieve good separation of the excess reagents from the analytes, and we have not noticed a significant deterioration of the column performance or lifespan.

**Figure 1.**
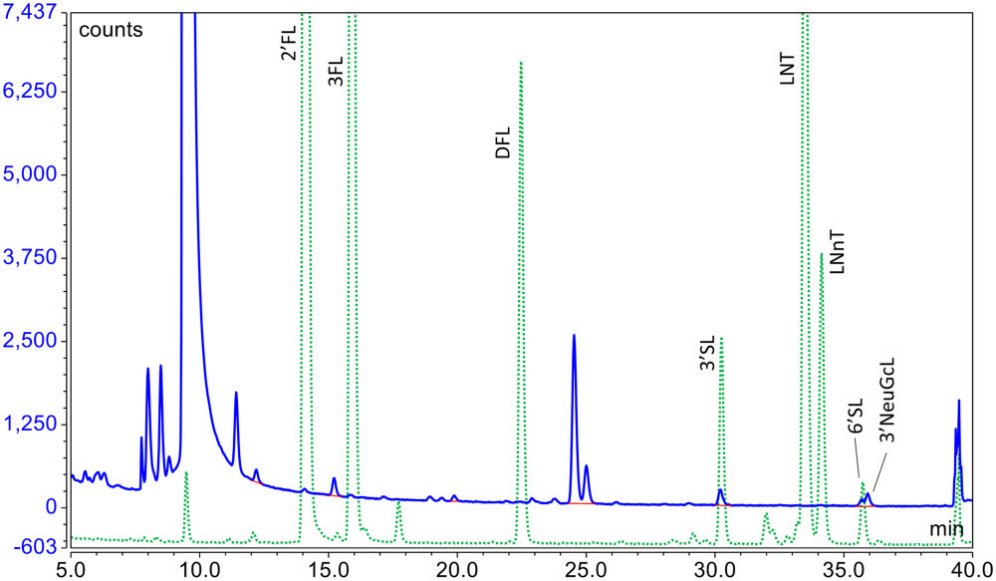
Overlay of chromatograms of HMO standards ( dotted line) and a goat-milk-based formula (solid line) showing the overlap of peaks for 6'SL and 3'NeuGcL at a column temperature of 60°C.

When analyzing human milk (15), the chromatography column was held at 55°C during the separation. However, under those conditions, lactose-3′-phosphate, a component of bovine milk and colostrum (31), elutes very close to the peak of 2′FL and may interfere with the analysis. Increasing the column temperature to 60°C improved the separation of those two components. However, at 60°C the peaks for two sialyllactoses, *N*-glycolylneuraminyl-3′-lactose (3′NeuGcL) and *N*-acetylneuraminyl-6′-lactose (6′SL), overlap (Figure 1). 3′NeuGcL is a minor component of cow milk, and in cow-milk-based formula, the concentration is too low to have an impact on the analysis of 6′SL. However, goat and sheep milk contain higher amounts of 3′NeuGcL, which impacts the 6′SL analysis, especially when 6′SL is at a low concentration. By increasing the column temperature to 75°C, 3′NeuGcL can be completely resolved from 6′SL. Thus, for analyzing such formula it is recommended to analyze all HMOs except 6′SL at 60°C, and then reinject at 75°C to determine the 6′SL.

This method has been developed and validated using a Waters BEH Glycan (1.7 µm, 2.1 × 150 mm) column; however, the newer Waters Premier Glycan BEH Amide may be preferable (avoiding the need for fetuin conditioning of the column). Other HILIC columns may be used for this analysis; however, conditions may need to be adapted depending on the column. We have found that varying the temperature in 5°C steps was often sufficient to find good separation conditions without needing to make other adjustments.

As mentioned in our previous publication (15), the quality of the oligosaccharides used for calibration is critical to achieve accurate results. The HMOs generally available from suppliers of laboratory chemicals are not well characterized (often determined solely on a chromatographic peak area, but without any description of the chromatographic conditions used). When such standards are used for calibration, the apparent HMO content can be significantly overestimated. To avoid this, it is best to use well-characterized standards, where the purity has been determined using several different methods, including (but not limited to) quantitative nuclear magnetic resonance spectroscopy (qNMR), and the moisture content has been determined by Karl Fischer. The standards should also be shipped and stored in a way to avoid further water absorption. Glycom/DSM offer well-characterized standards of analytical quality and were used for this study. Nevertheless, it may always be advisable to check the moisture content of standards (using Karl Fischer) before preparing calibration solutions. With growing interest in HMOs and their increased industrial production and use, additional suppliers and standards will hopefully be available in the future.

### Method Specificity

The main driver of specificity in this method is chromatography, although some specificity is also brought about by labeling the oligosaccharides with a specific tag (2AB) and detecting the fluorescence of the tag. As mentioned in the *Method Development* section, oligosaccharides naturally present in milk have the potential to interfere in the analysis. Lactose-3′-phosphate may interfere with the analysis of 2′FL. Using the conditions described in the method this will not normally be the case, but when using different columns it can be controlled by running a cow milk sample and a cow milk sample spiked with 2′FL; the 2′FL signal should not coelute with any signal in the non-spiked cow milk. 3′NeuGcL coelutes with 6′SL under the conditions described in the method. For cow-milk-based formula, this is not an issue since the concentrations of 3′NeuGcL are below the detection limit. However, when analyzing 6′SL in goat- or sheep-milk-based formula, it is recommended to perform an analysis at an elevated temperature (75°C) to resolve 6′SL from 3′NeuGcL.

**Figure 2.**
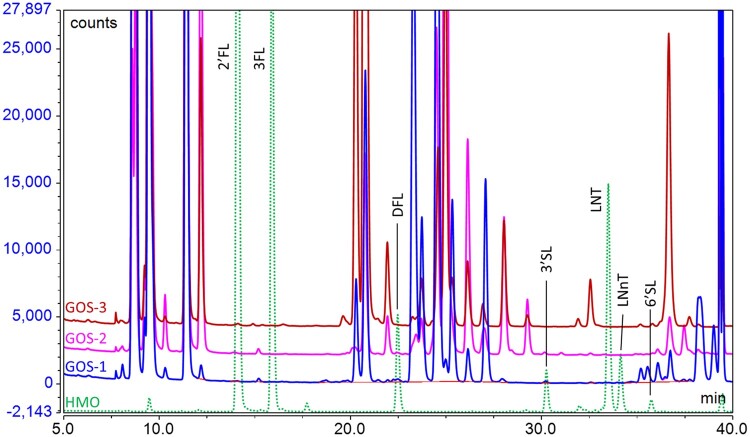
Overlay of chromatograms of HMO standards (dotted line) with three different GOS ingredients (solid lines) showing potential interference of GOS with 3'SL, 6'SL, and DFL.

**Figure 3.**
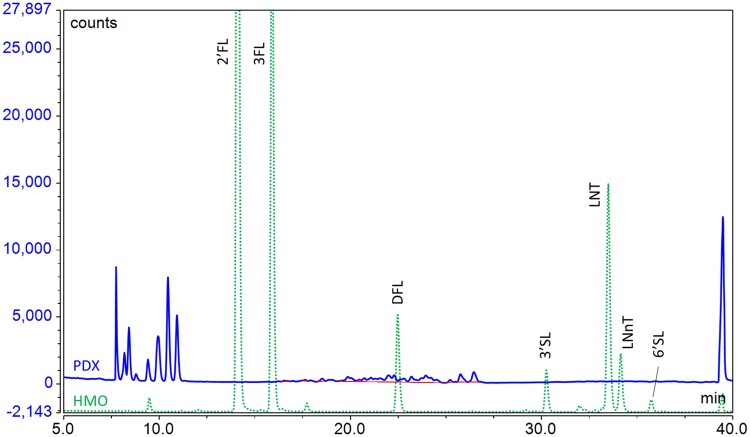
Overlay of chromatograms of HMO standards (dotted line) and a polydextrose (PDX) ingredient (solid line) showing potential interference of PDX with DFL.

Other oligosaccharides that may be added to infant formula also have the potential to interfere with the analysis. Maltodextrins may be added to formula as an energy source or as a carrier for other components (e.g., vitamins and minerals). Most of the peaks from maltodextrins do not interfere with the HMOs; however, maltotetraose elutes close to LNT and has the potential to interfere if present in high amounts. Maltodextrins are easily and specifically removed by treatment with amyloglucosidase (AMG), so treatment with this enzyme has been routinely included in the sample preparation. Beta-galactooligosaccharides (GOS) are a common component of many formulas, but the oligosaccharide profile changes between suppliers due to the different processes used to produce them (26, 32). We tested a number of different GOS ingredients to assess their impact and found that different GOS generated interferences with DFL, 3′SL, or 6′SL (Figure 2). To overcome this problem, an optional second sample preparation was introduced, in which a β-galactosidase was added along with the AMG to remove the GOS and the maltodextrins at the same time. However, the β-galactosidase also partially hydrolyzes LNT and LNnT, converting them both to lacto-*N*-triose (LNT-II). Therefore, it is necessary to determine LNT and LNnT following the procedure without β-galactosidase treatment; then, 3′SL, 6′SL, and DFL can be determined in the sample treated with β-galactosidase. GOS do not interfere with the analysis of 2′FL or 3FL, and neither are impacted with the β-galactosidase treatment and thus can be determined using either approach for sample preparation. Fructans (inulin or fructooligosaccharides) and polydextrose may also be added to formula as sources of nondigestible oligosaccharides. Since the fructans are predominantly non-reducing oligosaccharides or have a ketose at the reducing end, they are not labeled by the 2AB and thus do not interfere with the analysis. When a polydextrose ingredient was analyzed, a series of signals eluted early in the chromatogram before 2′FL and did not coelute with any of the signals of interest (Figure 3). However, an increased baseline noise was observed in the region of DFL (Figure 3); thus, the presence of polydextrose may be an issue if DFL is being analyzed close to the LoQ but otherwise should not be a problem.

**Figure 2022.07A.**
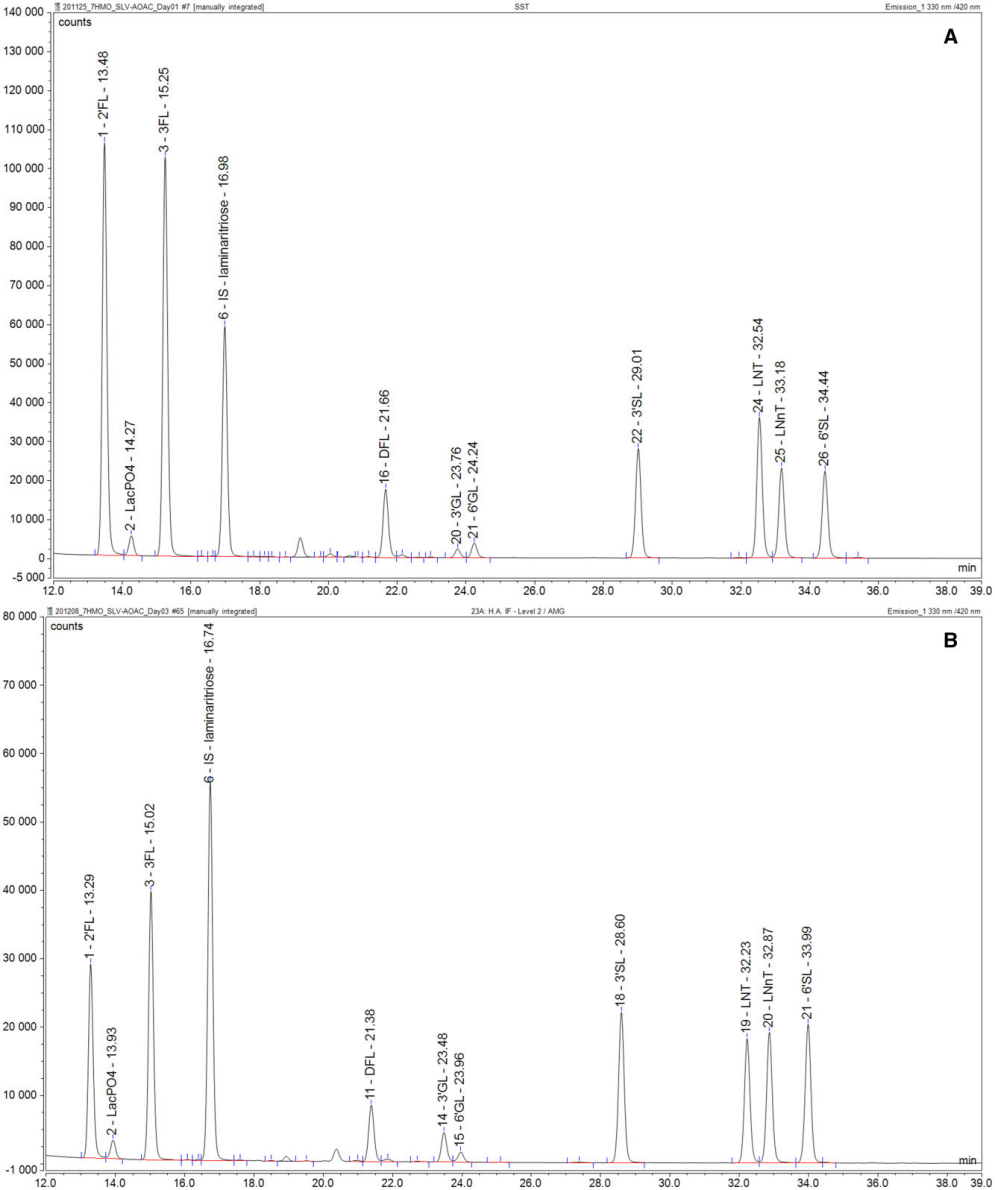
Typical chromatograms of seven HMOs. (A) Seven HMOs spiked in bovine milk, showing adequate separation between lactose phosphate and 2'FL. (B) HMOs in an infant formula with partially hydrolyzed proteins. 2′FL = 2′-fucosyllactose, LacPO4 = lactose phosphate, 3FL = 3-fucosyllactose, IS = internal standard, DFL = difucosyllactose, 3′GL = 3′-galactosyllactose (naturally present in bovine milk), 6′GL = 6′-galactosylactose (naturally present in bovine milk), 3′SL = 3′-sialyllactose, LNT = lacto-*N*-tetraose, LNnT = lacto-*N*-neotetraose, 6′SL = 6′sialyllactose.

### Calibration Fit

A linear model forced through zero was used to fit the calibration data for all HMOs (*see*Supplemental Figures 1–7), and the model appears to be a good fit. The relative residuals were calculated and plotted against the analyte concentration (*see*Supplemental Figures 1–7); for all but two points, the predicted concentration is within 5% of the actual concentration. The two points beyond that limit were both for 6′SL, one point at 3.73 µg/mL (-7.3%) and one point at 74.7 µg/mL (+5.9%). For 2′FL and 3FL, the plots of the relative residuals are not randomly distributed; there is a tendency for underestimation at low concentrations. However, the magnitude of the deviation is small enough that it does not have a significant impact on the analytical results.

### LoD and LoQ

The LoD and LoQ were estimated by spiking low levels of each HMO in the different matrixes as well as water. The level spiked represented about 10% of the desired LoQ specified in the SMPRs. The spiked samples were analyzed 10 times, and the mean and SD were combined to estimate the LoD and LoQ.

Three of the matrixes tested were based on cow milk, and thus contained inherent amounts of 3′SL and 6′SL that were too high to enable us to estimate a LoD and LoQ. Thus, for these two HMOs, LoD and LoQ were estimated only in a soy-based formula and in water. Based on the data for the other HMOs, it seems likely that the estimate for the soy-based formula should provide a good estimate for other types of formula.

In general, the estimated LoQs for all the HMOs meet the demands of the SMPR (Table 3). The only exception to this is DFL, for which there are some interferences in the chromatograms. We have identified that the laminaritriose internal standard used during these experiments contained a minor impurity which coeluted at the retention time of DFL. That impurity contributed to the increased LoQ for DFL; however, there are also matrix-based interferences that further increase the LoQ. The worst estimate for LoQ of DFL was 1.3 mg/100 g. Although this is higher than the desired LoQ of 1.2 mg/100 g, it remains below the lowest limit of the desired working range of 1.5 mg/100 g. The method may thus remain fit for purpose.

**Table 3.** Estimated LoD and LoQ for each HMO in different matrixes

Matrix	HMO	Mean, mg/100 g^a^	SD, mg/100 g^a^	LoD, mg/100 g^a^	LoQ, mg/100 g^a^
Cow milk formula with FOS	2′FL	0.305	0.014	0.35	0.44
Formula with partially hydrolyzed protein	2′FL	0.306	0.013	0.34	0.43
Formula with extensively hydrolyzed protein	2′FL	0.253	0.021	0.32	0.46
Soy-based formula	2′FL	0.328	0.027	0.42	0.61
Water	2’FL	0.349	0.016	0.40	0.51
Cow milk formula with FOS	3FL	0.253	0.011	0.28	0.36
Formula with partially hydrolyzed protein	3FL	0.243	0.008	0.27	0.33
Formula with extensively hydrolyzed protein	3FL	0.241	0.007	0.26	0.31
Soy-based formula	3FL	0.281	0.006	0.30	0.34
Water	3FL	0.278	0.013	0.32	0.41
Cow milk formula with FOS	DFL	0.830	0.040	0.95	1.2^b^
Formula with partially hydrolyzed protein	DFL	0.585	0.020	0.65	0.79
Formula with extensively hydrolyzed protein	DFL	0.407	0.093	0.69	1.3^b^
Soy-based formula	DFL	0.497	0.041	0.62	0.91
Water	DFL	0.195	0.012	0.23	0.31
Cow milk formula with FOS	LNT	0.090	0.013	0.13	0.22
Formula with partially hydrolyzed protein	LNT	0.092	0.011	0.13	0.21
Formula with extensively hydrolyzed protein	LNT	0.090	0.012	0.13	0.21
Soy-based formula	LNT	0.180	0.016	0.23	0.34
Water	LNT	0.093	0.022	0.16	0.31
Cow milk formula with FOS	LNnT	0.290	0.012	0.32	0.41
Formula with partially hydrolyzed protein	LNnT	0.284	0.010	0.13	0.21
Formula with extensively hydrolyzed protein	LNnT	0.299	0.019	0.36	0.49
Soy-based formula	LNnT	0.385	0.027	0.47	0.65
Water	LNnT	0.260	0.019	0.32	0.45
Soy-based formula	3′SL	0.089	0.010	0.12	0.19
Water	3′SL	0.092	0.013	0.13	0.22
Soy-based formula	6′SL	0.456	0.035	0.56	0.81
Water	6′SL	0.112	0.023	0.18	0.35

a Concentrations expressed as concentration found in reconstituted powders (25 g + 200 g water) or “ready-to-feed” liquid.

b The estimated LoQ is at or higher than the LoQ defined in the SMPR (1.2 mg/100 g for DFL).

### Accuracy/Trueness

Eight different blank matrixes were spiked at four different levels with the seven HMOs. Median recoveries were in the range of 90.9–109% across all HMOs and all matrixes (Tables 4–10), with a few exceptions. For 6′SL, LNT, and LNnT at the lowest spike level in the elemental formula, the spike recoveries were high (168% for 6′SL, 111% for LNnT, and 113% for LNT). For 6′SL, the issue could be resolved by increasing the column temperature to 75°C (as for goat-milk-based formula), which moves the 6′SL signal into an area of the chromatogram free from interference. In soy-based formula, the 6′SL was also overestimated at the lowest spike level (recovery of 121%). For all other matrixes and HMOs, the recoveries meet the requirements of the SMPRs. The mean recoveries were comparable to the median recoveries and are reported in Supplemental Tables 3–9. In general, the method appears to be suitable for the analysis of HMOs in most infant formula products, including those containing other nondigestible oligosaccharides and those containing probiotics. Nevertheless, the method may have limitations for the analysis of low levels of LNT, LNnT, and 6′SL in elemental formula and 6′SL in soy-based formula. Having seen the spike-recovery data for the elemental formula, we took a closer look at the matrix and estimated the contribution of the matrix to the apparent concentration of LNT, LNnT, and 6′SL in the product; by multiplying that by 10, we estimated the minimum level of each HMO that can be measured applying this method. This was estimated as 5 mg/100 g for LNnT, 2 mg/100 g for LNT, and 10 mg/100 g for 6′SL; likewise for soy-based formula, it was estimated that the minimum level of 6′SL that can be measured is around 4 mg/100 g.

**Table 4.** Spike recovery and precision estimates for 2'FL

Sample description	n	Matrix concn, mg/100 g^a^	Spike concn, mg/100 g^a^	Recovery, %	RSD_rob_(r), %	RSD_rob_(iR), %
Infant formula powder with FOS and GOS [1]	6 × 2	—	4.87	99.0	1.0	1.3
	6 × 2	—	20.7	103	0.7	1.2
	6 × 2	—	103	102	0.6	0.6
	6 × 2	—	511	101	0.3	0.5
Adult nutritional RTF with FOS and GOS	6 × 2	—	5.93	98.5	1.2	1.3
	6 × 2	—	20.2	100	0.8	2.6
	6 × 2	—	101	103	0.2	0.9
	6 × 2	—	504	101	0.6	1.2
Infant formula elemental powder	6 × 2	—	5.17	104	1.1	1.4
	6 × 2	—	20.5	104	1.0	0.8
	6 × 2	—	102	103	0.7	0.5
	6 × 2	—	499	102	0.6	1.2
Infant formula powder, soy-based	6 × 2	—	5.09	101	0.4	1.1
	6 × 2	—	20.3	105	0.5	1.5
	6 × 2	—	98.7	104	0.7	1.3
	6 × 2	—	494	103	0.5	1.2
Infant formula powder with partially hydrolyzed protein and probiotics	6 × 2	—	5.09	100	0.7	1.2
	6 × 2	—	20.3	103	0.9	1.3
	6 × 2	—	99.6	103	0.7	1.0
	6 × 2	—	495	103	0.7	1.6
Infant formula powder, goat milk based	6 × 2	—	5.09	104	0.7	0.9
	6 × 2	—	20.1	104	0.7	0.8
	6 × 2	—	98.8	104	0.6	0.9
	6 × 2	—	496	103	0.2	1.0
Infant formula RTF with FOS and GOS	6 × 2	—	5.08	100	0.4	2.5
	6 × 2	—	20.2	102	1.3	2.5
	6 × 2	—	98.7	102	0.6	2.8
	6 × 2	—	477	101	0.6	2.3
Infant formula powder with FOS and GOS [2]	7 × 2	—	15.2	103	0.7	1.8
	7 × 2	—	21.3	102	0.5	1.4
	7 × 2	—	30.7	102	1.5	1.5
	7 × 2	—	151	101	0.2	0.4
Commercial infant formula powder with GOS and 2′FL	6 × 2	80.8	—	—	1.0	1.6
Commercial infant formula powder with probiotic and 2′FL	6 × 2	19.7	—	—	1.1	1.9
Commercial infant formula RTF with 2′FL	6 × 2	24.6	—	—	0.9	1.8
Commercial infant formula powder with 2′FL and LNnT	6 × 2	86.4	—	—	0.7	1.8
Commercial infant formula powder with 2′FL, LNnT, and GOS	6 × 2	22.8	—	—	1.0	1.5
Pilot infant formula powder with 2′FL and LNnT	6 × 2	86.3	—	—	0.2	2.0
Pilot infant formula powder with GOS and HMOs (laboratory reference sample)	25 × 2	153^b^	—	—	0.9	2.4

a Concentrations reported on a “ready-to-feed” basis except ^b^reported as the concentration in the non-reconstituted powder.

**Table 5.** Spike recovery and precision estimates for 3FL

Sample description	n	Matrix concn, mg/100 g^a^	Spike concn, mg/100 g^a^	Recovery, %	RSD_rob_(r), %	RSD_rob_(iR), %
Infant formula powder with FOS and GOS [1]	6 × 2	—	4.22	101	0.6	1.6
	6 × 2	—	31.2	103	0.9	0.9
	6 × 2	—	82.4	103	0.5	1.2
	6 × 2	—	585	101	0.3	0.6
Adult nutritional RTF with FOS and GOS	6 × 2	—	4.49	100	1.0	2.4
	6 × 2	—	30.0	101	1.2	2.5
	6 × 2	—	84.5	103	0.5	1.0
	6 × 2	—	566	104	0.5	1.1
Infant formula elemental powder	6 × 2	—	3.72	103	1.0	1.2
	6 × 2	—	31.0	104	0.4	0.4
	6 × 2	—	83.3	102	0.5	0.5
	6 × 2	—	576	102	0.5	1.1
Infant formula powder, soy-based	6 × 2	—	4.03	103	0.6	2.2
	6 × 2	—	28.9	106	0.5	1.5
	6 × 2	—	98.5	105	0.5	0.7
	6 × 2	—	587	103	0.6	1.2
Infant formula powder with partially hydrolyzed protein and probiotics	6 × 2	—	4.03	101	0.4	0.9
	6 × 2	—	28.9	104	1.0	0.7
	6 × 2	—	99.4	104	0.6	0.8
	6 × 2	—	591	103	0.7	1.6
Infant formula powder, goat milk based	6 × 2	—	4.03	103	0.9	0.8
	6 × 2	—	28.8	104	0.6	0.9
	6 × 2	—	98.5	105	0.7	1.0
	6 × 2	—	584	103	0.2	0.6
Infant formula RTF with FOS and GOS	6 × 2	—	4.02	98.3	1.1	1.1
	6 × 2	—	28.7	101	1.1	1.9
	6 × 2	—	98.4	101	0.6	2.4
	6 × 2	—	600	100	0.5	1.6
Infant formula powder with FOS and GOS [2]	7 × 2	—	16.0	100	0.5	1.3
	7 × 2	—	48.6	101	0.8	1.5
	7 × 2	—	98.7	101	0.4	1.1
	7 × 2	—	147	100	0.6	1.7
Commercial infant formula powder with GOS and 2′FL	6 × 2	—	—	—	—	—
Commercial infant formula powder with probiotic and 2′FL	6 × 2	0.46	—	—	2.3	4.2
Commercial infant formula RTF with 2′FL	6 × 2	—	—	—	—	—
Commercial infant formula powder with 2′FL and LNnT	6 × 2	—	—	—	—	—
Commercial infant formula powder with 2′FL, LNnT, and GOS	6 × 2	—	—	—	—	—
Pilot infant formula powder with 2′FL and LNnT	6 × 2	—	—	—	—	—
Pilot infant formula powder with GOS and HMOs (laboratory reference sample)	25 × 2	—	—	—	—	—

a Concentrations reported on a “ready-to-feed” basis.

**Table 6.** Spike recovery and precision estimates for DFL

Sample description	n	Matrix concn, mg/100 g^a^	Spike concn, mg/100 g^a^	Recovery, %	RSD_rob_(r), %	RSD_rob_(iR), %
Infant formula powder with FOS and GOS [1]	6 × 2	—	1.59	97.8	0.7	2.2
	6 × 2	—	10.6	98.6	0.5	1.0
	6 × 2	—	50.9	103	0.6	1.6
	6 × 2	—	103	101	0.3	0.6
Adult nutritional RTF with FOS and GOS	6 × 2	—	1.66	90.9	2.5	6.2
	6 × 2	—	10.5	97.8	2.5	4.0
	6 × 2	—	52.6	97.8	0.7	0.7
	6 × 2	—	98.7	103	0.5	1.6
Infant formula elemental powder	6 × 2	—	1.54	108	0.9	7.5
	6 × 2	—	10.3	101	0.6	1.7
	6 × 2	—	50.3	102	0.5	1.1
	6 × 2	—	99.3	102	0.5	1.0
Infant formula powder, soy based	6 × 2	—	1.58	104	0.6	2.6
	6 × 2	—	9.98	102	0.7	2.3
	6 × 2	—	45.9	105	0.3	0.8
	6 × 2	—	96.3	103	1.0	1.2
Infant formula powder with partially hydrolyzed protein and probiotics	6 × 2	—	1.58	102	2.9	3.6
	6 × 2	—	10.0	102	0.4	1.5
	6 × 2	—	46.3	104	0.5	1.0
	6 × 2	—	97.1	103	0.8	1.2
Infant formula powder, goat milk based	6 × 2	—	1.60	97.8	1.3	3.2
	6 × 2	—	10.2	98.6	1.2	1.1
	6 × 2	—	46.7	103	0.5	1.1
	6 × 2	—	98.7	101	0.1	0.8
Infant formula RTF with FOS and GOS	6 × 2	—	1.83	95.6	0.9	5.6
	6 × 2	—	19.9	95.6	0.9	4.1
	6 × 2	—	50.1	102	1.2	3.0
	6 × 2	—	95.6	102	0.7	3.1
Infant formula powder with FOS and GOS [2]	7 × 2	—	2.99	94.3	0.40	1.3
	7 × 2	—	3.27	96.9	1.30	2.4
	7 × 2	—	5.18	94.4	1.70	5.4
	7 × 2	—	21.1	94.5	0.60	2.5
Commercial infant formula powder with GOS and 2′FL	6 × 2	—	—	—	—	—
Commercial infant formula powder with probiotic and 2′FL	6 × 2	<LoQ	—	—	—	—
Commercial infant formula RTF with 2′FL	6 × 2	—	—	—	—	—
Commercial infant formula powder with 2′FL and LNnT	6 × 2	<LoQ	—	—	—	—
Commercial infant formula powder with 2′FL, LNnT, and GOS	6 × 2	—	—	—	—	—
Pilot infant formula powder with 2′FL and LNnT	6 × 2	<LoQ	—	—	—	–
Pilot infant formula powder with GOS and HMOs (laboratory reference sample)	25 × 2	16.7^b^	—	—	1.3	2.3

a Concentrations reported on a “ready-to-feed” basis except ^b^ reported as the concentration in the non-reconstituted powder.

**Table 7.** Spike recovery and precision estimates for LNT

Sample description	n	Matrix concn, mg/100 g^a^	Spike concn, mg/100 g^a^	Recovery, %	RSD_rob_(r), %	RSD_rob_(iR), %
Infant formula powder with FOS and GOS [1]	6 × 2	—	2.01	101	0.8	2.5
	6 × 2	—	20.6	106	0.6	1.3
	6 × 2	—	147	103	0.6	0.5
	6 × 2	—	294	101	0.5	1.1
Adult nutritional RTF with FOS and GOS	6 × 2	—	2.24	101	4.2	3.6
	6 × 2	—	20.2	106	1.0	2.3
	6 × 2	—	147	106	0.5	0.5
	6 × 2	—	287	105	0.4	1.4
Infant formula elemental powder	6 × 2	—	1.96	113^c^	1.0	2.0
	6 × 2	—	21.3	106	0.4	1.1
	6 × 2	—	148	104	0.6	1.1
	6 × 2	—	290	104	0.4	1.4
Infant formula powder, soy based	6 × 2	—	2.01	107	1.0	1.1
	6 × 2	—	19.8	107	0.3	1.1
	6 × 2	—	119	107	0.5	1.1
	6 × 2	—	292	105	0.6	1.1
Infant formula powder with partially hydrolyzed protein and probiotics	6 × 2	—	2.02	99.8	1.0	1.4
	6 × 2	—	19.9	107	0.8	1.1
	6 × 2	—	120	106	0.6	0.8
	6 × 2	—	296	104	0.7	1.6
Infant formula powder, goat milk based	6 × 2	—	2.06	98.1	1.6	1.8
	6 × 2	—	20.2	103	0.9	0.8
	6 × 2	—	121	104	0.4	0.6
	6 × 2	—	294	103	0.2	1.0
Infant formula RTF with FOS and GOS	6 × 2	—	2.16	106	2.1	9.0
	6 × 2	—	21.2	104	1.2	5.3
	6 × 2	—	98.4	102	0.8	3.4
	6 × 2	—	304	100	0.7	1.6
Infant formula powder with FOS and GOS [2]	7 × 2	—	7.06	106	1.1	3.4
	7 × 2	—	11.3	104	0.3	3.1
	7 × 2	—	28.1	102	0.9	2.5
	7 × 2	—	50.9	100	0.2	1.9
Commercial infant formula powder with GOS and 2′FL	6 × 2	—	—	—	—	—
Commercial infant formula powder with probiotic and 2′FL	6 × 2	—	—	—	—	—
Commercial infant formula RTF with 2′FL	6 × 2	—	—	—	—	—
Commercial infant formula powder with 2′FL and LNnT	6 × 2	—	—	—	—	—
Commercial infant formula powder with 2′FL, LNnT, and GOS	6 × 2	—	—	—	—	—
Pilot infant formula powder with 2′FL and LNnT	6 × 2	—	—	—	—	—
Pilot infant formula powder with GOS and HMOs (laboratory reference sample)	25 × 2	52.5^b^	—	—	1.4	2.8

a Concentrations reported on a “ready-to-feed” basis except ^b^ reported as the concentration in the non-reconstituted powder

c Performance does not meet SMPR.

**Table 8.** Spike recovery and precision estimates for LNnT

Sample description	n	Matrix concn, mg/100 g^a^	Spike concn, mg/100 g^a^	Recovery, %	RSD_rob_(r), %	RSD_rob_(iR), %
Infant formula powder with FOS and GOS [1]	6 × 2	—	6.08	100	0.5	0.7
	6 × 2	—	23.1	102	0.5	1.1
	6 × 2	—	73.9	102	0.5	1.0
	6 × 2	—	103	101	0.3	1.2
Adult nutritional RTF with FOS and GOS	6 × 2	—	5.56	99.1	0.7	5.6
	6 × 2	—	22.2	102	1.2	3.3
	6 × 2	—	74.8	104	0.6	1.1
	6 × 2	—	103	104	0.4	1.2
Infant formula elemental powder	6 × 2	—	5.35	111^c^	0.8	1.5
	6 × 2	—	23.4	106	0.4	1.2
	6 × 2	—	75.6	103	1.4	2.2
	6 × 2	—	103	104	0.4	1.2
Infant formula powder, soy based	6 × 2	—	5.19	102	0.8	2.0
	6 × 2	—	21.3	105	0.3	1.6
	6 × 2	—	72.2	106	0.7	1.3
	6 × 2	—	101	104	0.9	1.1
Infant formula powder with partially hydrolyzed protein and probiotics	6 × 2	—	5.19	99.9	1.0	1.1
	6 × 2	—	21.3	104	0.8	1.2
	6 × 2	—	72.8	105	1.0	1.4
	6 × 2	—	104	104	0.7	2.0
Infant formula powder, goat milk based	6 × 2	—	5.41	102	0.7	1.2
	6 × 2	—	21.3	102	1.3	1.1
	6 × 2	—	72.2	106	0.4	0.6
	6 × 2	—	101	105	0.7	1.6
Infant formula RTF with FOS and GOS	6 × 2	—	5.18	103	1.1	3.3
	6 × 2	—	21.2	102	1.1	3.5
	6 × 2	—	72.1	105	0.7	2.7
	6 × 2	—	106	104	0.5	1.6
Infant formula powder with FOS and GOS [2]	7 × 2	—	10.2	104	0.5	2.5
	7 × 2	—	20.9	105	0.3	1.6
	7 × 2	—	40.8	106	0.9	2.6
	7 × 2	—	77.3	104	0.2	2.9
Commercial infant formula powder with GOS and 2′FL	6 × 2	—	—	—	—	—
Commercial infant formula powder with probiotic and 2′FL	6 × 2	—	—	—	—	—
Commercial infant formula RTF with 2′FL	6 × 2	—	—	—	—	—
Commercial infant formula powder with 2′FL and LNnT	6 × 2	43.6	—	—	1.4	3.1
Commercial infant formula powder with 2′FL, LNnT, and GOS	6 × 2	10.7	—	—	1.1	2.0
Pilot infant formula powder with 2′FL and LNnT	6 × 2	43.7	—	—	0.1	1.5
Pilot infant formula powder with GOS and HMOs (laboratory reference sample)	25 × 2	95.8^b^	—	—	1.0	2.0

a Concentrations reported on a “ready-to-feed” basis except ^b^ reported as the concentration in the non-reconstituted powder.

c Performance does not meet SMPR.

**Table 9.** Spike recovery and precision estimates for 3'SL

Sample description	n	Matrix concn, mg/100 g^a^	Spike concn, mg/100 g^a^	Recovery, %	RSD_rob_(r), %	RSD_rob_(iR), %
Infant formula powder with FOS and GOS [1]	6 × 2	4.10	—	—	0.5	1.6
	6 × 2	4.10	17.7	105	0.8	1.4
	6 × 2	4.10	73.9	102	0.3	0.7
	6 × 2	4.10	144	101	0.4	1.1
Adult nutritional RTF with FOS and GOS	6 × 2	0.95	—	—	3.3	10.0
	6 × 2	0.95	3.66	104	1.4	4.0
	6 × 2	0.95	22.3	104	1.1	3.4
	6 × 2	0.95	74.6	105	0.8	0.6
	6 × 2	0.95	139	106	0.6	1.1
Infant formula elemental powder	6 × 2	—	1.04	103	1.0	4.0
	6 × 2	—	26.1	105	1.1	1.6
	6 × 2	—	75.0	102	0.6	0.8
	6 × 2	—	144	102	0.6	1.1
Infant formula powder, soy based	6 × 2	—	1.50	104	1.0	1.4
	6 × 2	—	21.0	105	0.5	0.8
	6 × 2	—	67.3	105	0.5	0.8
	6 × 2	—	144	103	0.4	1.1
Infant formula powder with partially hydrolyzed protein and probiotics	6 × 2	2.04	—	—	1.8	1.7
	6 × 2	2.04	21.1	104	0.6	0.8
	6 × 2	2.04	67.9	104	0.6	1.2
	6 × 2	2.04	139	103	0.7	1.4
Infant formula powder, goat milk based	6 × 2	1.29	—	—	2.4	2.4
	6 × 2	1.29	21.0	103	0.9	1.4
	6 × 2	1.29	67.3	105	0.7	1.2
	6 × 2	1.29	143	103	0.3	0.9
Infant formula RTF with FOS and GOS	6 × 2	5.15	—	—	1.2	5.6
	6 × 2	5.15	3.20	102	0.7	3.1
	6 × 2	5.15	25.1	106	0.9	2.8
	6 × 2	5.15	79.1	103	2.3	2.7
	6 × 2	5.15	142	102	3.1	2.1
Infant formula powder with FOS and GOS [2]	7 × 2	4.56	—	—	0.5	2.4
	7 × 2	6.86	1.17	99.1	0.3	1.7
	7 × 2	5.97	2.19	100	1.4	2.6
	7 × 2	6.33	4.05	101	0.6	1.4
	7 × 2	4.56	14.0	101	0.9	2.0
Commercial infant formula powder with GOS and 2′FL	6 × 2	3.41	—	—	0.6	1.8
Commercial infant formula powder with probiotic and 2′FL	6 × 2	1.93	—	—	1.4	2.7
Commercial infant formula RTF with 2′FL	6 × 2	5.75	—	—	0.9	2.5
Commercial infant formula powder with 2′FL and LNnT	6 × 2	1.74	—	—	1.5	2.6
Commercial infant formula powder with 2′FL, LNnT, and GOS	6 × 2	6.31	—	—	0.9	3.3
Pilot infant formula powder with 2′FL and LNnT	6 × 2	5.59	—	—	0.7	2.9
Pilot infant formula powder with GOS and HMOs (laboratory reference sample)	25 × 2	98.0^b^	—	—	0.7	2.1

a Concentrations reported on a “ready-to-feed” basis except ^b^ reported as the concentration in the non-reconstituted powder.

**Table 10.** Spike recovery and precision estimates for 6'SL

Sample description	n	Matrix concn, mg/100 g^a^	Spike concn, mg/100 g^a^	Recovery, %	RSD_rob_(r), %	RSD_rob_(iR), %
Infant formula powder with FOS and GOS [1]	6 × 2	0.82	—	—	2.6	3.4
	6 × 2	0.82	21.2	106	0.9	2.1
	6 × 2	0.82	78.4	105	0.4	2.9
	6 × 2	0.82	148	102	0.3	1.9
Adult nutritional RTF with FOS and GOS	6 × 2	—	2.23	109	2.9	3.4
	6 × 2	—	24.4	105	0.5	3.7
	6 × 2	—	80.2	107	0.7	1.9
	6 × 2	—	142	107	0.5	1.8
Infant formula elemental powder	6 × 2	—	1.77	168^b^	0.9	4.5
	6 × 2	—	24.6	106	1.0	2.6
	6 × 2	—	78.1	105	0.6	2.3
	6 × 2	—	142	104	0.5	1.8
Infant formula elemental powder (75°C elution)	6 × 2	—	1.77	103	1.4	3.9
	6 × 2	—	24.6	105	1.0	2.2
	6 × 2	—	78.1	102	0.7	2.7
	6 × 2	—	142	103	1.3	4.2
Infant formula powder, soy based	6 × 2	—	1.94	121^c^	0.4	6.9
	6 × 2	—	22.4	108	0.2	1.4
	6 × 2	—	71.8	107	0.7	1.7
	6 × 2	—	148	102	0.4	1.7
Infant formula powder with partially hydrolyzed protein and probiotics	6 × 2	0.56	—	—	3.8	5.5
	6 × 2	0.56	1.48	103	2.0	1.4
	6 × 2	0.56	22.5	106	1.2	1.8
	6 × 2	0.56	72.5	106	0.6	0.6
	6 × 2	0.56	144	104	0.7	0.9
Infant formula powder, goat milk based	6 × 2	0.83	—	—	1.9	3.8
	6 × 2	0.83	22.9	109	1.5	3.1
	6 × 2	0.83	73.4	106	0.3	1.9
	6 × 2	0.83	144	103	0.5	2.6
Infant formula powder, goat milk based (75°C elution)	6 × 2	0.90	—	—	1.2	2.8
	6 × 2	0.90	22.9	103	0.7	3.1
	6 × 2	0.90	73.4	103	0.5	1.1
	6 × 2	0.90	144	103	0.7	2.7
Infant formula RTF with FOS and GOS	6 × 2	1.15	—	—	1.8	7.9
	6 × 2	1.15	2.28	103	0.4	3.9
	6 × 2	1.15	24.6	105	0.7	2.9
	6 × 2	1.15	84.4	104	1.4	2.4
	6 × 2	1.15	147	102	0.1	2.0
Infant formula powder with FOS and GOS [2]	7 × 2	1.35	—	—	2.4	4.6
	7 × 2	1.31	3.17	103	0.7	5.2
	7 × 2	1.16	8.39	103	0.9	3.5
	7 × 2	1.35	13.5	102	0.6	2.7
	7 × 2	0.83	19.3	101	0.7	3.5
Commercial infant formula powder with GOS and 2′FL	6 × 2	0.72	—	—	1.5	2.7
Commercial infant formula powder with probiotic and 2′FL	6 × 2	0.52	—	—	4.1	6.4
Commercial infant formula RTF with 2′FL	6 × 2	1.31	—	—	1.2	4.2
Commercial infant formula powder with 2′FL and LNnT	6 × 2	0.39	—	—	2.7	10
Commercial infant formula powder with 2′FL, LNnT, and GOS	6 × 2	1.31	—	—	1.6	6.2
Pilot infant formula powder with 2′FL and LNnT	6 × 2	1.09	—	—	0.7	2.9
Pilot infant formula powder with GOS and HMOs (laboratory reference sample)	25 × 2	42.4^b^	—	—	1.0	3.0

a Concentrations reported on a “ready-to-feed” basis except ^b^ reported as the concentration in the non-reconstituted powder.

c Performance does not meet SMPR.

**Table 2022.07A.** Scheme for preparation of working standard solutions

Calibration level	Vol. of stock solution, µL [**E**(**a**–**g**)]							Final vol., mL
	2′FL	3FL	DFL	3′SL	6′SL	LNT	LNnT	
1	50	50	50	40	40	40	50	50
2	110	110	70	75	75	70	60	10
3	110	110	70	80	80	70	60	5
4	170	170	110	130	130	110	90	5
5	240	240	150	180	180	150	130	5
6	300	300	200	240	240	200	170	5

**Table 2022.07B.** Example recipes to prepare 2AB labeling reagent

Maximum no. of tests	DMSO–acetic acid (70:30)		2AB, 0.35 mol/L–picoline borane, 1.0 mol/L in DMSO–acetic acid [70:30] solution		
	DMSO, mL	Glacial acetic acid, mL	DMSO–acetic acid, mL (70:30)	2AB, mg	Picoline borane, mg
50	4.70	2.0	6.00	286 ± 5	625 ± 5
100	11.6	5.0	12.5	600 ± 10	1300 ± 15
250	23.3	10.0	30.0	1430 ± 15	3120 ± 15

**Table 2022.07C.** Recommended dilution depending on expected HMO concentration of “ready-to-feed” or reconstituted product

HMO	Standard levels (dilute 20 g in 50 mL)^a^	High levels (dilute 5 g in 50 mL)^a^
2′FL	0.5–150 mg/100 g	2–600 mg/100 g
3FL	0.5–150 mg/100 g	2–600 mg/100 g
DFL	1–40 mg/100 g	4–150 mg/100 g
LNT	0.4–70 mg/100 g	2–300 mg/100 g
LNnT	0.5–50 mg/100 g	2–200 mg/100 g
3′SL	0.2–50 mg/100 g	1–200 mg/100 g
6′SL	1–50 mg/100 g	4–200 mg/100 g

a Concentrations apply to i) “ready-to-feed” liquids “as is,” ii) reconstituted powders (25 g + 200 g water), iii) liquid concentrates diluted 1:1 by weight.

**Table 2022.07D.** Chromatographic gradient

Time, min	Flow, mL/min	A, %^a^	B, %^b^
0.0	0.500	90.0	10.0
4.00	0.500	90.0	10.0
38.00	0.500	80.1	19.9
38.50	0.500	20.0	80.0
41.50	0.500	20.0	80.0
42.00	0.500	90.0	10.0
52.00	0.500	90.0	10.0

a Eluent A = Acetonitrile.

b Eluent B = Ammonium formate (50 mM, pH 4.4).

### Precision

The eight matrixes, each spiked with the seven HMOs at four different concentrations, were analyzed on at least six different days in duplicate and were used to estimate the method repeatability (r) and intermediate reproducibility (iR). In addition to the spiked matrixes, seven additional formulas from commercial production or pilot trials containing at least one of the HMOs were analyzed on at least six different days in duplicate. Samples were analyzed by four different analysts, using three different instruments and three different columns (same model, different serial numbers).

The SMPRs require the relative repeatability (RSD(r)) of the method to be below 5% for all HMOs at all concentrations (Table 1). The data collected during the SLV indicate that the method achieves this, with the robust RSD(r) for all the HMOs in all matrixes covering the range of 0.1–4.2% (Tables 4–10). The SMPRs require the reproducibility (RSD(R)) of the method to be below 10% for all HMOs at all concentrations (Table 1). Reproducibility cannot be measured during a SLV; however, the robust intermediate reproducibility (RSD_rob_(iR)) can be used to estimate if the method is likely to meet the reproducibility requirements. The RSD_rob_(iR) lies between 0.4 and 10% (Tables 4–10) at the concentration ranges defined in the SMPRs, indicating a good chance of success if the method performance is tested in a multi-laboratory trial. Supplemental Tables 3–9 report comparable RSD(r) and RSD(iR) data calculated using classic statistics.

## Conclusions

The method described is suitable for the determination of seven HMOs (2′FL, 3FL, DFL, LNT, LNnT, 3′SL, and 6′SL) in most infant formula within the concentration ranges specified in the SMPRs. However, there may be some limitations if the method is applied for the analysis of low levels of HMOs in elemental formula or for the analysis of 6′SL in soy-based formula.

The repeatability and recoveries of the method generally meet the requirements of the SMPRs, with the exception of low levels of DFL, LNT, and LNnT in elemental formula and 6′SL in soy-based formula. Other oligosaccharides that may be found in infant formula such as maltodextrins, FOS, and GOS do not interfere with the analysis. Polydextrose may interfere with the accurate determination of DFL if DFL is present at low concentrations.

## Supplemental Information

Supplemental information is available on the *J. AOAC Int*. website.

## Funding

Funding support for this article was provided by the Société des Produits Nestlé S.A.

## Conflict of Interest

None declared.

## Supplementary Material

qsae001_Supplementary_Data
