# Supplementary material for: Determination of Seven Human Milk Oligosaccharides (HMOs) in Infant Formula and Adult Nutritionals: First Action 2022.07
Source: J AOAC Int. 2024 Jan 13;107(2):286–302. doi: 10.1093/jaoacint/qsae001 (PMC10907138; doi:10.1093/jaoacint/qsae001)
Supplement: qsae001_Supplementary_Data [file qsae001_supplementary_data.pdf]

Supplementary Table 1: Spike levels used for estimation of LoD and LoQ

	HMO spike, mg/100g[a]						
	2’FL	3FL	DFL	3’SL	LNT	LNnT	6’SL
Cow milk formula with FOS	0.328	0.271	0.100	0.000 [b]	0.113	0.335	0.000 [b]
Formula with partially hydrolyzed protein	0.328	0.271	0.100	0.000 [b]	0.113	0.335	0.000 [b]
Formula with extensively hydrolyzed protein	0.326	0.270	0.100	0.000 [b]	0.112	0.333	0.000 [b]
Soy based formula	0.318	0.263	0.097	0.113	0.110	0.325	0.150
Water	0.327	0.271	0.100	0.101	0.113	0.335	0.131

[a] Concentrations expressed as concentration found in reconstituted powders (25 + 200g water) or “ready-to-feed” liquid .
[b] The milk-based formula contain native concentrations of 3’SL and 6’SL, which are too high for estimating LoD and LoQ using low level spikes.

Supplementary Table 2: Matrices and spike levels used for spike-recovery experiments

Matrix	HMO	Spike level 1, mg/100g[a]	Spike level 2, mg/100g[a]	Spike level 3, mg/100g[a]	Spike level 4, mg/100g[a]
Adult nutritional RTF with FOS and GOS	2’FL	5.93	20.2	101	504
	3FL	4.49	30.0	84.5	566
	DFL	1.66	10.5	52.6	98.7
	LNT	2.24	20.2	147	287
	LNnT	5.56	22.2	74.8	103
	3’SL	3.66	22.3	74.6	139
	6’SL	2.23	24.4	80.2	142
Infant formula elemental powder[b]	2’FL	5.17	20.5	102	499
	3FL	3.72	31.0	83.3	576
	DFL	1.54	10.3	50.3	99.3
	LNT	1.96	21.3	148	290
	LNnT	5.35	23.4	75.6	103
	3’SL	1.04	26.1	75.0	144
	6’SL	1.77	24.6	78.1	142
Infant formula powder with FOS and GOS [1][b,c]	2’FL	4.87	20.7	103	511
	3FL	4.22	31.2	82.4	585
	DFL	1.59	10.6	50.9	103
	LNT	2.01	20.6	147	294
	LNnT	6.08	23.1	73.9	103
	3’SL	0.0	17.7	73.9	144
	6’SL	0.0	21.2	78.4	148
Infant formula powder soy-based	2’FL	5.09	20.3	98.7	494
	3FL	4.03	28.9	98.5	587
	DFL	1.58	9.98	45.9	96.3
	LNT	2.01	19.8	119	292
	LNnT	5.19	21.3	72.2	101
	3’SL	1.50	21.0	67.3	144
	6’SL	1.94	22.4	71.8	148
Infant formula powder with partially hydrolyzed protein and probiotics	2’FL	5.09	20.3	99.6	495
	3FL	4.03	28.9	99.4	591
	DFL	1.58	10.0	46.3	97.1
	LNT	2.02	19.9	120	296
	LNnT	5.19	21.3	72.8	104
	3’SL	0.00	21.1	67.9	139
	6’SL	1.48	22.5	72.5	144
Infant formula powder, goat milk based	2’FL	5.09	20.1	98.8	496
	3FL	4.03	28.8	98.5	584
	DFL	1.60	10.2	46.7	98.7

Supplementary Table 2: Matrices and spike levels used for spike-recovery experiments

Matrix	HMO	Spike level 1, mg/100g[a]	Spike level 2, mg/100g[a]	Spike level 3, mg/100g[a]	Spike level 4, mg/100g[a]
	LNT	2.06	20.2	121	294
	LNnT	5.41	21.3	72.2	101
	3'SL	0.00	21.0	67.3	143
	6'SL	0.00	22.9	73.4	144
Infant formula RTF with FOS and GOS	2'FL	5.08	20.2	98.7	477
	3FL	4.02	28.7	98.4	600
	DFL	1.83	19.9	50.1	95.6
	LNT	2.16	21.2	98.4	304
	LNnT	5.18	21.2	72.1	106
	3'SL	3.20	25.1	79.1	142
	6'SL	2.28	24.6	84.4	147
Infant formula powder with FOS and GOS [2][c]	2'FL	15.2	21.3	30.7	151
	3FL	147	98.7	48.6	16.0
	DFL	2.99	3.27	5.18	21.1
	LNT	7.06	11.3	28.1	50.9
	LNnT	10.2	20.9	40.8	77.3
	3'SL	1.17	2.19	4.05	14.0
	6'SL	3.17	8.39	13.5	19.3

[a] Concentrations expressed as concentration found in reconstituted powders (25 g + 200g water) or "ready-to-feed" liquid.
[b] Matrix from the SPIFAN 2 matrices kit.
[c] Infant formula powders with GOS and FOS [1] and [2] are two different products and contain different sources of GOS.

Supplementary Table 3: Spike-recovery and precision estimates for 2'FL (Classic Statistics)

Sample description	*n*	*Matrix concn[a], mg/100g*	*Spike concn[a], mg/100g*	*Recovery, %*	*RSD(r), %*	*RSD(iR), %*
Infant formula powder with FOS and GOS [1]	6×2	–	4.87	99.0	0.8	1.1
	6×2	–	20.7	104	0.7	0.9
	6×2	–	103	102	0.7	1.1
	6×2	–	511	101	0.5	0.9
Adult nutritional RTF with FOS and GOS	6×2	–	5.93	97.9	1.2	2.5
	6×2	–	20.2	101	0.6	1.9
	6×2	–	101	103	0.6	0.9
	6×2	–	504	102	0.9	1.3
Infant formula elemental powder	6×2	–	5.17	104	0.9	1.6
	6×2	–	20.5	104	1.3	1.1
	6×2	–	102	102	0.6	1.7
	6×2	–	499	102	0.9	1.3
Infant formula powder soy based	6×2	–	5.09	101	0.5	1.1
	6×2	–	20.3	105	0.5	1.1
	6×2	–	98.7	104	0.5	0.9
	6×2	–	494	103	0.4	1.1
Infant formula powder with partially hydrolyzed protein and probiotics	6×2	–	5.09	100	0.9	1.2
	6×2	–	20.3	103	0.7	1.0
	6×2	–	99.6	102	3.0	3.2
	6×2	–	495	103	0.4	1.0
Infant formula powder goat milk based	6×2	–	5.09	103	0.6	0.8
	6×2	–	20.1	104	0.8	0.8
	6×2	–	98.8	104	0.6	0.8
	6×2	–	496	103	0.3	1.1
Infant formula RTF with FOS and GOS	6×2	–	5.08	100	0.8	2.1
	6×2	–	20.2	102	1.0	2.2
	6×2	–	98.7	102	0.7	2.0
	6×2	–	477	101	0.4	1.5
Infant formula powder with FOS and GOS [2]	7×2	–	15.2	103	0.9	1.6
	7×2	–	21.3	102	0.6	1.3
	7×2	–	30.7	102	3.0	2.8
	7×2	–	151	100	0.2	1.2
Commercial infant formula powder with GOS and 2'FL	6×2	80.8	–	–	0.8	1.5
Commercial infant formula powder with probiotic and 2'FL	6×2	19.7	–	–	1.0	2.1
Commercial infant formula RTF with 2'FL	6×2	24.6	–	–	0.7	1.7
Commercial infant formula powder with 2'FL and LNnT	6×2	86.4	–	–	2.5	2.4
Commercial infant formula powder with 2'FL, LNnT and GOS	6×2	22.8	–	–	1.6	1.4
Pilot infant formula powder with 2'FL and LNnT	6×2	86.3	–	–	0.2	1.7
Pilot infant formula powder with GOS and HMOs (lab reference sample)	25×2	153*	–	–	0.9	2.1

[a] *Concentrations reported on a "ready-to-feed" basis except * reported as the concentration in the non-reconstituted powder.*

Supplementary Table 4: Spike-recovery and precision estimates for 3FL (Classic Statistics)

Sample description	*n*	*Matrix concn[a], mg/100g*	*Spike concn[a], mg/100g*	*Recovery, %*	*RSD(r), %*	*RSD(iR), %*
Infant formula powder with FOS and GOS [1]	6×2	–	4.22	101	0.7	1.2
	6×2	–	31.2	103	0.6	0.7
	6×2	–	82.4	102	0.9	1.2
	6×2	–	585	101	0.5	0.7
Adult nutritional RTF with FOS and GOS	6×2	–	4.49	100	0.8	1.5
	6×2	–	30.0	101	1.2	2.4
	6×2	–	84.5	103	0.6	0.8
	6×2	–	566	104	0.9	1.3
Infant formula elemental powder	6×2	–	3.72	103	0.6	1.8
	6×2	–	31.0	104	1.0	1.3
	6×2	–	83.3	101	0.6	1.9
	6×2	–	576	102	0.9	1.3
Infant formula powder soy based	6×2	–	4.03	103	0.7	1.5
	6×2	–	28.9	106	0.5	1.1
	6×2	–	98.5	105	0.3	0.7
	6×2	–	587	103	0.6	1.0
Infant formula powder with partially hydrolyzed protein and probiotics	6×2	–	4.03	101	1.1	1.0
	6×2	–	28.9	104	0.7	0.7
	6×2	–	99.4	103	3.0	3.3
	6×2	–	591	102	0.4	1.5
Infant formula powder goat milk based	6×2	–	4.03	103	0.8	0.7
	6×2	–	28.8	104	0.8	0.9
	6×2	–	98.5	104	0.7	0.9
	6×2	–	584	104	0.3	0.8
Infant formula RTF with FOS and GOS	6×2	–	4.02	99.6	1.0	4.5
	6×2	–	28.7	102	1.0	0.9
	6×2	–	98.4	102	0.7	1.1
	6×2	–	600	100	0.5	1.0
Infant formula powder with FOS and GOS [2]	7×2	–	16.0	100	0.3	1.3
	7×2	–	48.6	102	3.0	1.3
	7×2	–	98.7	101	0.6	2.9
	7×2	–	147	101	0.8	1.4
Commercial infant formula powder with GOS and 2'FL	6×2	–	–	–	–	–
Commercial infant formula powder with probiotic and 2'FL	6×2	0.46	–	–	1.7	3.3
Commercial infant formula RTF with 2'FL	6×2	–	–	–	–	–
Commercial infant formula powder with 2'FL and LNnT	6×2	–	–	–	–	–
Commercial infant formula powder with 2'FL, LNnT and GOS	6×2	–	–	–	–	–
Pilot infant formula powder with 2'FL and LNnT	6×2	–	–	–	–	–
Pilot infant formula powder with GOS and HMOs (lab reference sample)	25×2	–	–	–	–	–

[a] *Concentrations reported on a "ready-to-feed" basis*

Supplementary Table 5: Spike-recovery and precision estimates for DFL (Classic Statistics)

Sample description	n	Matrix concn[a], mg/100g	Spike concn[a], mg/100g	Recovery, %	RSD(r), %	RSD(iR), %
Infant formula powder with FOS and GOS [1]	6×2	–	1.59	97.6	1.4	1.8
	6×2	–	10.6	98.6	0.6	0.8
	6×2	–	50.9	103	0.5	1.1
	6×2	–	103	101	0.6	0.9
Adult nutritional RTF with FOS and GOS	6×2	–	1.66	91.8	2.1	6.0
	6×2	–	10.5	97.9	2.0	3.5
	6×2	–	52.6	101	0.6	0.5
	6×2	–	98.7	103	0.9	1.2
Infant formula elemental powder	6×2	–	1.54	108	1.0	6.3
	6×2	–	10.3	101	1.8	1.8
	6×2	–	50.3	102	0.6	1.6
	6×2	–	99.3	102	0.9	1.2
Infant formula powder soy based	6×2	–	1.58	104	0.5	4.1
	6×2	–	9.98	102	0.5	2.3
	6×2	–	45.9	105	0.4	0.7
	6×2	–	96.3	103	0.7	1.0
Infant formula powder with partially hydrolyzed protein and probiotics	6×2	–	1.58	104	2.3	7.6
	6×2	–	10.0	102	0.6	1.5
	6×2	–	46.3	103	3.0	3.1
	6×2	–	97.1	104	0.6	1.9
Infant formula powder goat milk based	6×2	–	1.60	98.6	2.0	5.0
	6×2	–	10.2	98.7	1.0	1.1
	6×2	–	46.7	103	0.5	0.8
	6×2	–	98.7	102	0.3	0.8
Infant formula RTF with FOS and GOS	6×2	–	1.83	95.5	1.0	3.9
	6×2	–	19.9	95.7	0.8	2.6
	6×2	–	50.1	103	1.7	3.3
	6×2	–	95.6	102	1.2	2.7
Infant formula powder with FOS and GOS [2]	7×2	–	2.99	97.5	1.3	8.4
	7×2	–	3.27	99.4	1.6	10.6
	7×2	–	5.18	97.1	1.3	6.7
	7×2	–	21.1	95.0	0.5	2.1
Commercial infant formula powder with GOS and 2’FL	6×2	–	–	–	–	–
Commercial infant formula powder with probiotic and 2’FL	6×2	<LoQ	–	–	–	–
Commercial infant formula RTF with 2’FL	6×2	–	–	–	–	–
Commercial infant formula powder with 2’FL and LNnT	6×2	<LoQ	–	–	–	–
Commercial infant formula powder with 2’FL, LNnT and GOS	6×2	–	–	–	–	–
Pilot infant formula powder with 2’FL and LNnT	6×2	<LoQ	–	–	–	–
Pilot infant formula powder with GOS and HMOs (lab reference sample)	25×2	16.7*	–	–	0.0	3.4

[a] Concentrations reported on a “ready-to-feed” basis except * reported as the concentration in the non-reconstituted powder.

Supplementary Table 6: Spike-recovery and precision estimates for LNT (Classic Statistics)

Sample description	*n*	*Matrix concn[a], mg/100g*	*Spike concn[a], mg/100g*	*Recovery, %*	*RSD(r), %*	*RSD(iR), %*
Infant formula powder with FOS and GOS [1]	6×2	–	2.01	102	2.2	4.4
	6×2	–	20.6	107	1.5	1.3
	6×2	–	147	103	0.8	1.0
	6×2	–	294	102	0.6	1.1
Adult nutritional RTF with FOS and GOS	6×2	–	2.24	102	4.1	3.4
	6×2	–	20.2	105	0.8	3.0
	6×2	–	147	106	0.7	0.9
	6×2	–	287	105	0.9	1.4
Infant formula elemental powder	6×2	–	1.96	112[b]	0.9	2.8
	6×2	–	21.3	106	1.1	1.4
	6×2	–	148	103	0.7	1.9
	6×2	–	290	104	0.9	1.4
Infant formula powder soy based	6×2	–	2.01	106	0.8	1.1
	6×2	–	19.8	107	0.4	0.9
	6×2	–	119	107	0.4	0.8
	6×2	–	292	105	0.5	1.1
Infant formula powder with partially hydrolyzed protein and probiotics	6×2	–	2.02	100	0.9	1.1
	6×2	–	19.9	107	0.7	0.8
	6×2	–	120	105	3.0	3.2
	6×2	–	296	104	0.4	1.2
Infant formula powder goat milk based	6×2	–	2.06	98.1	1.2	1.3
	6×2	–	20.2	103	0.9	0.9
	6×2	–	121	104	0.6	0.8
	6×2	–	294	103	0.3	1.1
Infant formula RTF with FOS and GOS	6×2	–	2.16	106	1.8	5.7
	6×2	–	21.2	105	1.0	4.5
	6×2	–	98.4	103	0.7	3.1
	6×2	–	304	101	0.4	2.6
Infant formula powder with FOS and GOS [2]	7×2	–	7.06	105	1.2	3.1
	7×2	–	11.3	103	0.6	2.6
	7×2	–	28.1	103	3.0	3.3
	7×2	–	50.9	100	0.2	2.1
Commercial infant formula powder with GOS and 2'FL	6×2	–	–	–	–	–
Commercial infant formula powder with probiotic and 2'FL	6×2	–	–	–	–	–
Commercial infant formula RTF with 2'FL	6×2	–	–	–	–	–
Commercial infant formula powder with 2'FL and LNnT	6×2	–	–	–	–	–
Commercial infant formula powder with 2'FL, LNnT and GOS	6×2	–	–	–	–	–
Pilot infant formula powder with 2'FL and LNnT	6×2	–	–	–	–	–
Pilot infant formula powder with GOS and HMOs (lab reference sample)	25×2	52.5*	–	–	1.1	2.5

*[a] Concentrations reported on a "ready-to-feed" basis except * reported as the concentration in the non-reconstituted powder.*
[b] Performance does not meet SMPR.

Supplementary Table 7: Spike-recovery and precision estimates for LNnT (Classic Statistics)

Sample description	n	Matrix concn[a], mg/100g	Spike concn[a], mg/100g	Recovery, %	RSD(r), %	RSD(iR), %
Infant formula powder with FOS and GOS [1]	6×2	–	6.08	100	0.5	0.7
	6×2	–	23.1	102	0.6	0.9
	6×2	–	73.9	102	0.8	0.9
	6×2	–	103	101	0.7	1.0
Adult nutritional RTF with FOS and GOS	6×2	–	5.56	99.6	1.5	4.4
	6×2	–	22.2	101	1.2	2.9
	6×2	–	74.8	104	0.6	0.9
	6×2	–	103	104	1.1	1.3
Infant formula elemental powder	6×2	–	5.35	110	0.6	2.4
	6×2	–	23.4	105	1.2	1.6
	6×2	–	75.6	103	0.9	2.1
	6×2	–	103	104	1.1	1.3
Infant formula powder soy based	6×2	–	5.19	102	0.7	1.8
	6×2	–	21.3	104	0.4	1.3
	6×2	–	72.2	106	0.5	1.0
	6×2	–	101	104	0.7	0.9
Infant formula powder with partially hydrolyzed protein and probiotics	6×2	–	5.19	100	1.0	0.9
	6×2	–	21.3	104	0.6	1.1
	6×2	–	72.8	105	3.0	3.0
	6×2	–	104	104	0.5	2.0
Infant formula powder goat milk based	6×2	–	5.41	102	0.8	1.1
	6×2	–	21.3	102	1.1	0.8
	6×2	–	72.2	106	0.5	0.7
	6×2	–	101	105	0.4	1.1
Infant formula RTF with FOS and GOS	6×2	–	5.18	102	1.5	3.9
	6×2	–	21.2	103	1.1	2.9
	6×2	–	72.1	105	0.9	2.4
	6×2	–	106	104	0.7	1.7
Infant formula powder with FOS and GOS [2]	7×2	–	10.2	105	0.2	3.2
	7×2	–	20.9	105	0.1	2.4
	7×2	–	40.8	107	1.3	3.4
	7×2	–	77.3	105	0.3	2.7
Commercial infant formula powder with GOS and 2’FL	6×2	–	–	–	–	–
Commercial infant formula powder with probiotic and 2’FL	6×2	–	–	–	–	–
Commercial infant formula RTF with 2’FL	6×2	–	–	–	–	–
Commercial infant formula powder with 2’FL and LNnT	6×2	43.6	–	–	2.5	3.2
Commercial infant formula powder with 2’FL, LNnT and GOS	6×2	10.7	–	–	1.6	2.3
Pilot infant formula powder with 2’FL and LNnT	6×2	43.7	–	–	0.1	2.2
Pilot infant formula powder with GOS and HMOs (lab reference sample)	25×2	95.8*	–	–	1.0	2.0

[a] *Concentrations reported on a “ready-to-feed” basis except * reported as the concentration in the non-reconstituted powder.*

Supplementary Table 8: Spike-recovery and precision estimates for 3'SL (Classic Statistics)

Sample description	*n*	*Matrix concn[a], mg/100g*	*Spike concn[a], mg/100g*	*Recovery, %*	*RSD(r), %*	*RSD(iR), %*
Infant formula powder with FOS and GOS [1]	6×2	4.10	–	–	0.5	1.1
	6x2	4.10	17.7	104	0.5	1.1
	6×2	4.10	73.9	103	0.3	1.1
	6×2	4.10	144	101	0.3	0.8
Adult nutritional RTF with FOS and GOS	6×2	0.95	–	–	2.3	10.4
	6×2	0.95	3.66	104	1.3	3.4
	6×2	0.95	22.3	103	1.0	2.6
	6x2	0.95	74.6	104	0.9	1.0
	6×2	0.95	139	106	0.9	1.2
Infant formula elemental powder	6×2	–	1.04	104	1.9	2.7
	6×2	–	26.1	105	1.9	1.7
	6×2	–	75.0	101	0.7	1.8
	6×2	–	144	102	0.9	1.2
Infant formula powder soy based	6×2	–	1.50	105	0.9	1.4
	6×2	–	21.0	105	0.5	0.7
	6×2	–	67.3	106	0.4	0.7
	6×2	–	144	103	0.4	1.1
Infant formula powder with partially hydrolyzed protein and probiotics	6×2	2.04	–	–	1.4	1.3
	6x2	2.04	21.1	104	0.6	0.8
	6×2	2.04	67.9	104	3.0	3.2
	6×2	2.04	139	103	0.6	1.7
Infant formula powder goat milk based	6×2	1.29	–	–	1.7	1.9
	6x2	1.29	21.0	103	0.9	1.0
	6×2	1.29	67.3	105	0.7	0.9
	6×2	1.29	143	103	0.4	0.9
Infant formula RTF with FOS and GOS	6×2	5.15	–	–	0.8	3.2
	6×2	5.15	3.20	102	1.0	1.9
	6x2	5.15	25.1	106	1.1	1.7
	6×2	5.15	79.1	104	1.8	2.1
	6×2	5.15	142	102	1.3	1.9
Infant formula powder with FOS and GOS [2]	7×2	4.56	–	–	0.4	1.5
	7×2	6.86	1.17	99.1	0.7	1.4
	7×2	5.97	2.19	101	1.1	2.0
	7×2	6.33	4.05	102	0.4	1.6
	7×2	4.56	14.0	101	0.7	1.7
Commercial infant formula powder with GOS and 2'FL	6×2	3.41	–	–	0.7	2.7
Commercial infant formula powder with probiotic and 2'FL	6×2	1.93	–	–	1.1	2.6
Commercial infant formula RTF with 2'FL	6×2	5.75	–	–	0.8	2.3
Commercial infant formula powder with 2'FL and LNnT	6×2	1.74	–	–	1.6	4.3
Commercial infant formula powder with 2'FL, LNnT and GOS	6×2	6.31	–	–	1.7	4.3
Pilot infant formula powder with 2'FL and LNnT	6×2	5.59	–	–	0.4	2.4
Pilot infant formula powder with GOS and HMOs (lab reference sample)	25×2	98.0*	–	–	0.0	2.4

*[a] Concentrations reported on a "ready-to-feed" basis except * reported as the concentration in the non-reconstituted powder.*

Supplementary Table 9: Spike-recovery and precision estimates for 6'SL (Classic Statistics)

Sample description	*n*	*Matrix concn[a], mg/100g*	*Spike concn[a], mg/100g*	*Recovery, %*	*RSD(r), %*	*RSD(iR), %*
Infant formula powder with FOS and GOS [1]	6×2	0.82	–	–	3.9	1.0
	6x2	0.82	21.2	106	0.6	1.5
	6×2	0.82	78.4	105	0.3	2.0
	6×2	0.82	148	102	0.3	1.6
Adult nutritional RTF with FOS and GOS	6×2	–	2.23	110	2.3	5.2
	6×2	–	24.4	105	1.3	2.8
	6×2	–	80.2	107	0.9	1.4
	6×2	–	142	107	0.4	1.8
Infant formula elemental powder	6×2	–	1.77	142[b]	0.9	33.4
	6×2	–	24.6	106	1.9	2.3
	6×2	–	78.1	104	0.7	2.4
	6×2	–	142	104	0.9	1.5
Infant formula elemental powder (75°C elution)	6×2	–	1.77	103	2.5	4.0
	6×2	–	24.6	106	1.8	3.2
	6×2	–	78.1	102	0.7	2.0
	6×2	–	142	103	1.1	2.6
Infant formula powder soy based	6×2	–	1.94	121[b]	0.6	5.4
	6×2	–	22.4	108	0.3	1.7
	6×2	–	71.8	107	0.4	1.6
	6×2	–	148	102	0.4	1.5
Infant formula powder with partially hydrolyzed protein and probiotics	6×2	0.56	–	–	2.8	5.5
	6x2	0.56	1.48	103	1.4	1.4
	6×2	0.56	22.5	106	0.8	1.8
	6×2	0.56	72.5	105	2.9	0.6
	6×2	0.56	144	105	0.6	0.9
Infant formula powder goat milk based	6×2	0.83	–	–	1.5	3.8
	6x2	0.83	22.9	109	1.2	2.6
	6×2	0.83	73.4	105	0.6	2.1
	6×2	0.83	144	103	0.5	2.0
Infant formula powder goat milk based (75°C elution)	6×2	0.90	–	–	2.4	3.4
	6x2	0.90	22.9	104	0.9	2.1
	6×2	0.90	73.4	105	0.5	2.1
	6×2	0.90	144	102	0.6	2.3
Infant formula RTF with FOS and GOS	6×2	1.15	–	–	1.5	5.7
	6×2	1.15	2.28	104	0.9	3.9
	6x2	1.15	24.6	106	1.1	3.1
	6×2	1.15	84.4	105	1.9	3.3
	6×2	1.15	147	104	1.3	3.6
Infant formula powder with FOS and GOS [2]	7×2	1.35	–	–	1.8	4.6
	7×2	1.31	3.17	104	0.8	4.0
	7×2	1.16	8.39	103	1.0	3.5
	7×2	1.35	13.5	102	0.3	3.4
	7×2	0.83	19.3	102	0.4	3.7
Commercial infant formula powder with GOS and 2'FL	6×2	0.72	–	–	1.2	3.9
Commercial infant formula powder with probiotic and 2'FL	6×2	0.52	–	–	3.0	6.0
Commercial infant formula RTF with 2'FL	6×2	1.31	–	–	2.4	3.3
Commercial infant formula powder with 2'FL and LNnT	6×2	0.39	–	–	2.8	8.0
Commercial infant formula powder with 2'FL, LNnT and GOS	6×2	1.31	–	–	2.3	5.4
Pilot infant formula powder with 2'FL and LNnT	6×2	1.09	–	–	2.3	3.8
Pilot infant formula powder with GOS and HMOs (lab reference sample)	25×2	42.4*	–	–	0.0	4.0

*[a] Concentrations reported on a "ready-to-feed" basis except * reported as the concentration in the non-reconstituted powder.*
[b] Performance does not meet SMPR.

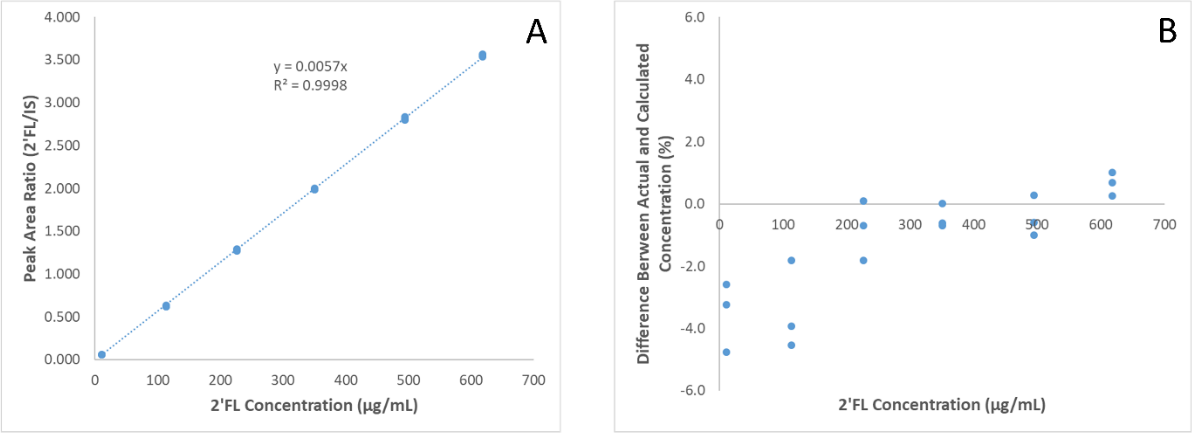

Supplementary Figure 1: Calibration fit for 2'FL. A: Calibration curve. B: Plot of relative residuals.

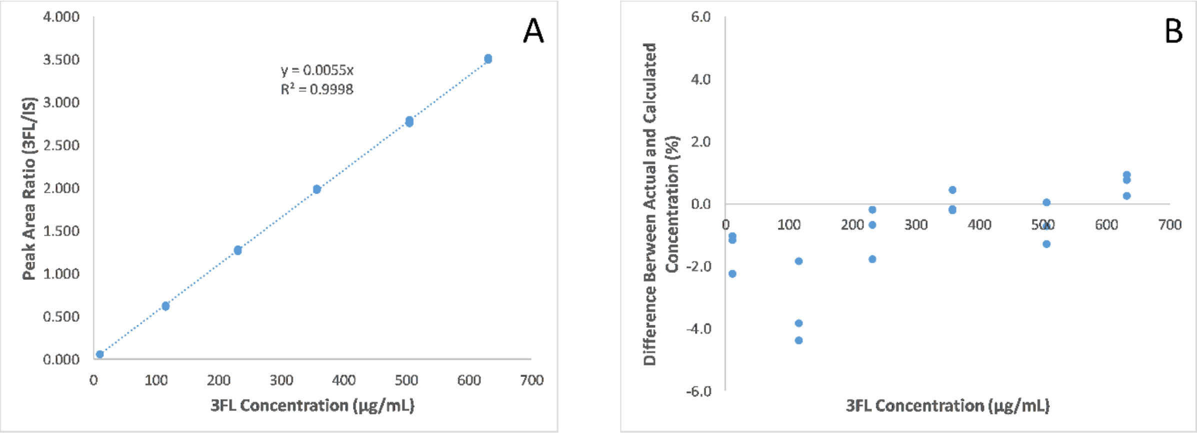

Supplementary Figure 2: Calibration fit for 3FL. A: Calibration curve. B: Plot of relative residuals.

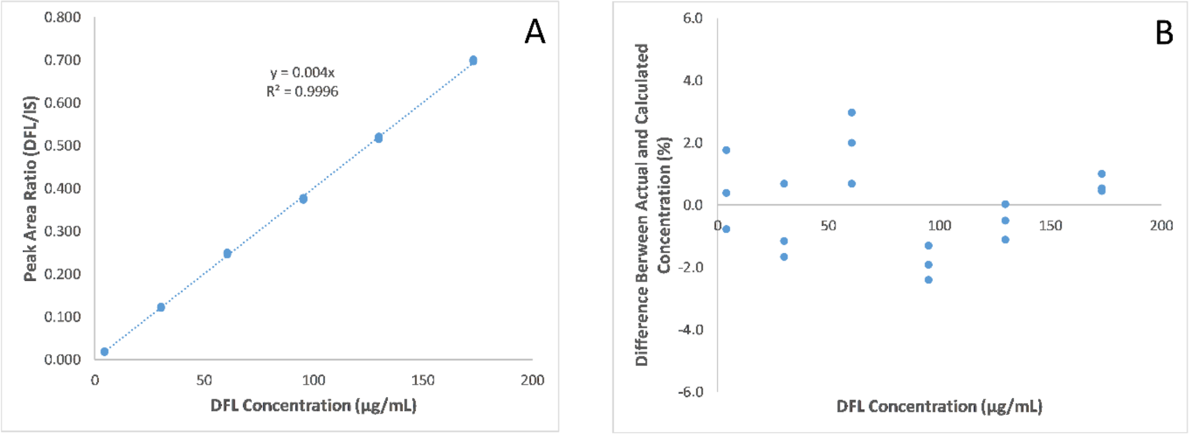

Supplementary Figure 3: Calibration fit for DFL. A: Calibration curve. B: Plot of relative residuals

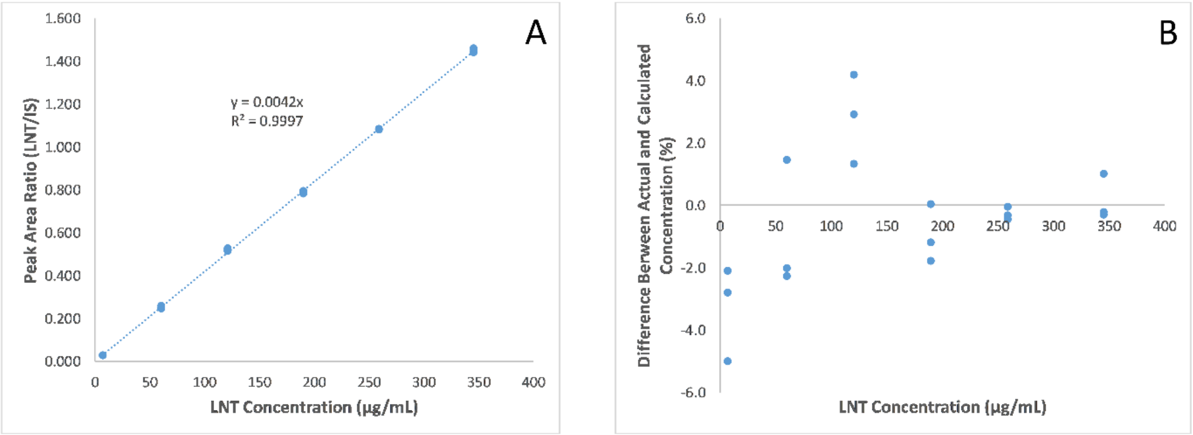

Supplementary Figure 4: Calibration fit for LNT. A: Calibration curve. B: Plot of relative residuals.

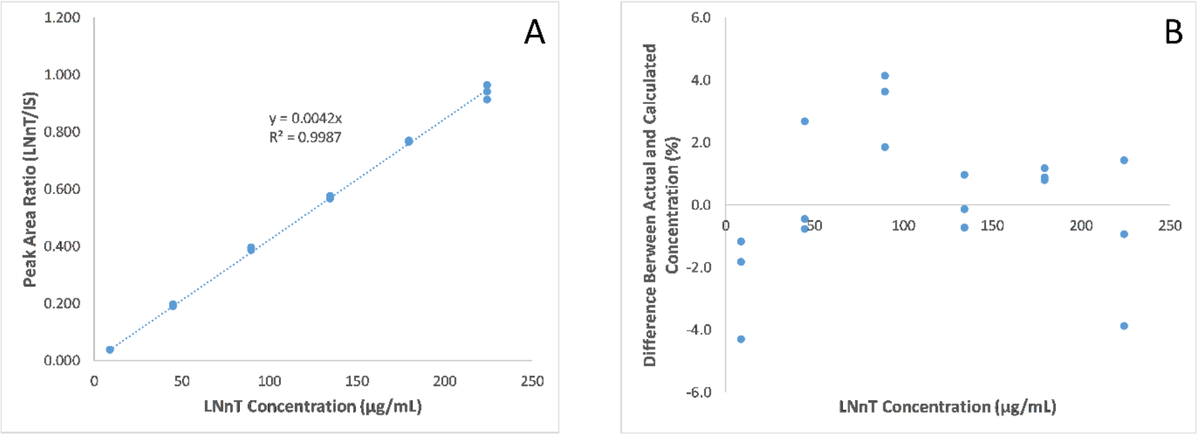

Supplementary Figure 5: Calibration fit for LNnT. A: Calibration curve. B: Plot of relative residuals.

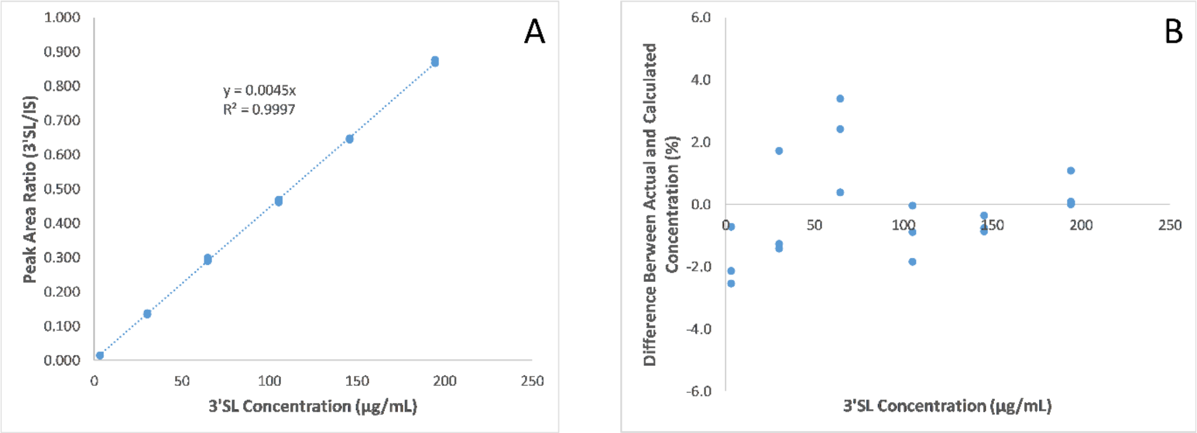

Supplementary Figure 6: Calibration fit for 3’SL. A: Calibration curve. B: Plot of relative residuals.

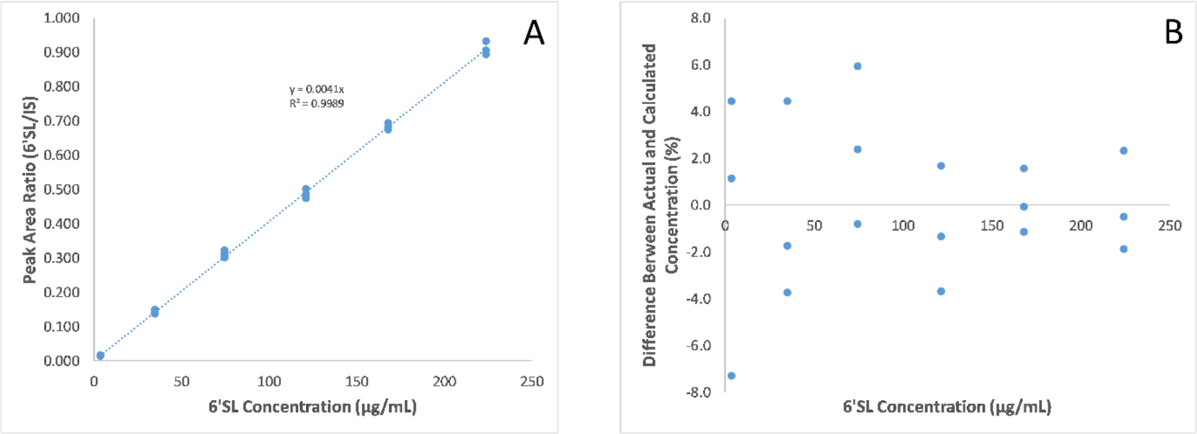

Supplementary Figure 7: Calibration fit for 6'SL. A: Calibration curve. B: Plot of relative residuals.
